# The AR/miR-221/IGF-1 pathway mediates the pathogenesis of androgenetic alopecia

**DOI:** 10.7150/ijbs.80481

**Published:** 2023-06-26

**Authors:** Kaitao Li, Yang Sun, Shizhao Liu, Yi Zhou, Qian Qu, Gaofeng Wang, Jin Wang, Ruosi Chen, Zhexiang Fan, Bingcheng Liu, Yuning Li, Xiaoyan Mao, Zhiqi Hu, Yong Miao

**Affiliations:** Department of Plastic and Aesthetic Surgery, Nanfang Hospital, Southern Medical University, 1838 North Guangzhou AV, Guangzhou, Guangdong Province, 510515, China.

**Keywords:** androgenetic alopecia, miR-221, androgen receptor, IGF-1, dihydrotestosterone

## Abstract

Androgenetic alopecia (AGA) affects more than half of the adult population worldwide and is primarily caused by the binding of dihydrotestosterone (DHT) to androgen receptors (AR). However, the mechanisms by which AR affects hair follicles remain unclear. In our study, we found that miR-221 significantly suppressed hair growth and the proliferation of dermal papilla cells (DPCs) and dermal sheath cells (DSCs) in AGA patients. Interestingly, miR-221 and AR were mainly co-located in the same part of the hair follicle. Mechanistic analysis revealed that AR directly promoted the transcription of miR-221, which in turn suppressed IGF-1 expression, leading to the inactivation of the MAPK pathway in DPCs and the PI3K/AKT pathway in DSCs. In AGA patients, miR-221 expression was positively correlated with AR expression and negatively correlated with IGF-1 expression. Our findings indicate that miR-221, as a direct target of AR, plays a crucial role in the pathogenesis of AGA, making it a novel biomarker and potential therapeutic target for treating AGA.

## Introduction

Androgenetic alopecia (AGA), also known as male pattern baldness, is the predominant cause of hair loss among men. It affects over 50% of men by the age of 50 and typically manifests in the temples, vertex, and mid-frontal scalp region^1^. AGA is characterized by a shortened anagen phase, prolonged telogen phase, and miniaturization of hair follicles^2^,^3^. The pathogenesis of AGA is primarily driven by dihydrotestosterone (DHT), which is converted from testosterone via the 5-α reductase enzyme^4^. In susceptible hair follicles, DHT binds to the androgen receptor (AR) resulting in the activation of genes responsible for the transformation of anagen to catagen and the conversion of large terminal follicles to miniaturized follicles^5^,^6^. AGA patients have elevated levels of 5-α reductase enzyme and AR in frontal hair follicles compared to occipital follicles^7^. However, the direct target genes of AR in susceptible hair follicles have not been comprehensively elucidated.

The hair follicle is a complex mini-organ comprising both dermal and epidermal compartments, and the interactions between these two compartments are essential for hair follicle growth, morphogenesis, and regeneration^8^. The dermal compartment of the hair follicle is composed of the dermal papilla and dermal sheath. The dermal papilla is located at the bottom of the hair follicle and is surrounded by epithelial matrix cells^9^. Dermal papilla cells (DPCs) are known to provide instructive signals required to activate epithelial progenitors, thereby initiating hair follicle regeneration^10^. The dermal sheath is located at the outermost border of hair follicles and contains progenitor cells that maintain and repopulate the DPCs^11^. Cell immigration and emigration between the dermal sheath and dermal papilla compartments during the hair cycle are necessary for cell survival and hair growth^12^. Therefore, DPCs and DSCs is a key compartment regulating hair follicle growth.

Insulin-like growth factor 1 (IGF-1) plays a critical role in regulating the hair cycle, including controlling hair shaft differentiation and tissue remodeling, and serving as a key regulator of mitogenic and morphogenetic processes during hair follicle development^13^,^14^. As such, IGF-1 has emerged as a promising therapeutic target for hair loss conditions, including AGA and alopecia areata^15^,^16^. The downstream pathways of IGF-1 have been extensively studied in various cell types, including lung carcinoma^17^, skeletal muscle^18^, and myoblast cell^19^. Specifically, the PI3K/AKT pathway has been identified as a critical signaling axis responsible for mediating the effects of IGF-1 in these contexts. Similarly, the MAPK pathway is regulated upstream by IGF-1 in a variety of cell types, including myoblast cells^20^, hepatocytes^21^, and dental pulp stem cells^22^. Despite these findings, the precise extent to which IGF-1 regulates hair follicle biology via the PI3K/AKT and MAPK pathways remains an open question.

MicroRNAs (miRNAs) are a class of short non-coding RNAs that regulate gene expression post-transcriptionally^23^. Typically, miRNAs bind to the 3'-UTR (untranslated region) of their target mRNAs, resulting in the repression of protein production through mRNA destabilization and translational silencing^24^. Multiple miRNAs may regulate a particular mRNA, while a single miRNA may target many mRNAs^25^. Moreover, the transcription of miRNAs can be initiated and regulated by transcription factors that bind to their promoter regions^26^. It is estimated that miRNAs potentially regulate at least 20%-30% of all human protein-coding coding genes that are closely involved in various biological processes, including cell growth, apoptosis, proliferation, inflammation, and immune responses^27^. Although several studies have demonstrated a correlation between miRNAs and AGA^28^-^30^, the comprehensive molecular mechanisms underlying this relationship remain incompletely understood.

**Scheme 1:**
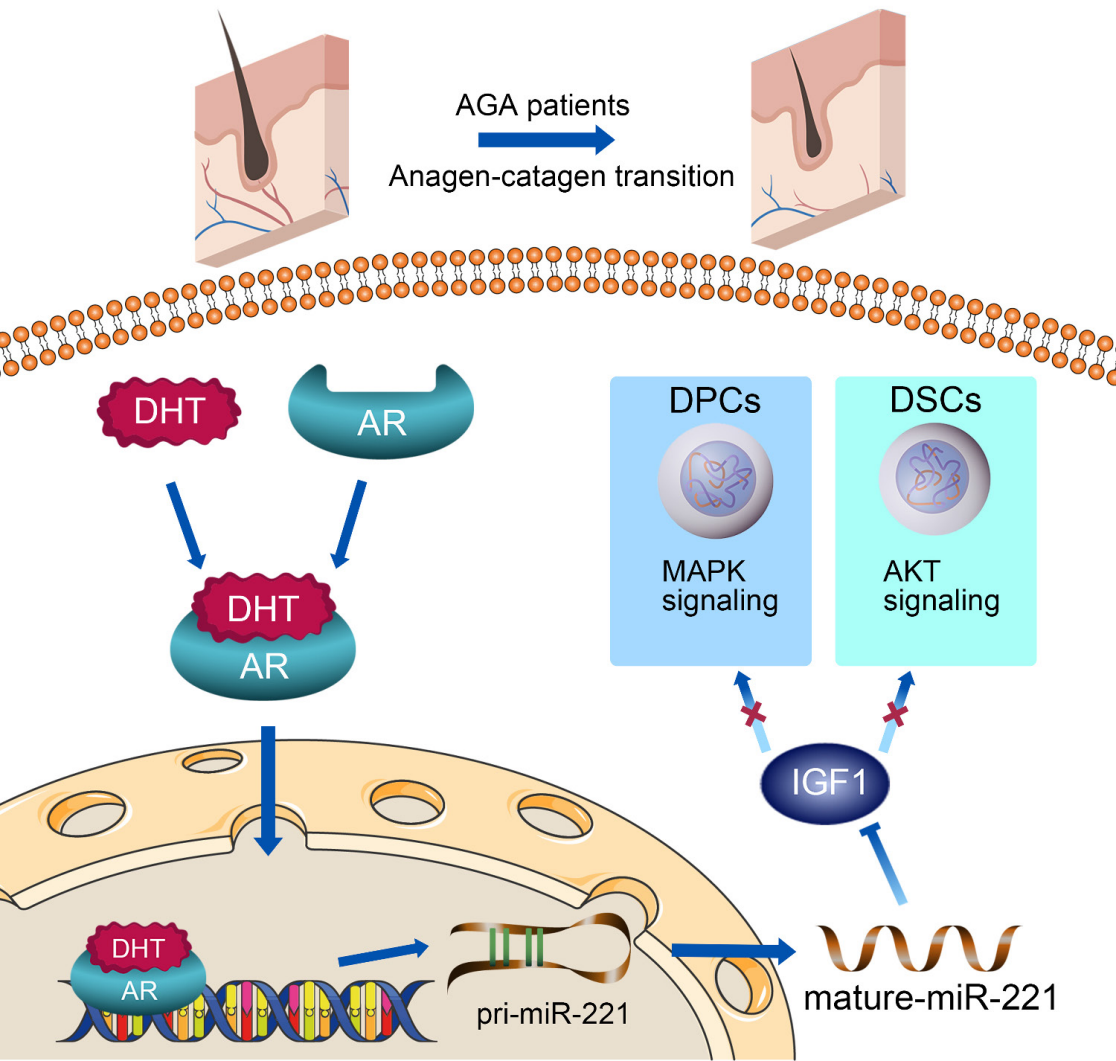
A schematic representation for the AR/miR-221/IGF1 axis regulating MAPK pathway and PI3K/AKT pathway.

In this study, miR-221 was found to be significantly upregulated in balding hair follicles, thereby contributing to the suppression of hair growth and proliferation. The transcription of miR-221 was directly promoted by AR in combination with DHT in DPCs and DSCs. Mechanistic analysis revealed that miR-221 could directly suppress IGF-1 expression, leading to the inactivation of the MAPK pathway in DPCs and the PI3K/AKT pathway in DSCs (**Scheme 1**). Notably, miR-221 expression is positively correlated with both AR and IGF-1 expression in patients with AGA. These findings highlight the potential value of miR-221 as a novel biomarker and therapeutic target for AGA.

## Results

### miR-221 inhibits the proliferation of DPCs and DSCs

**Figure 1:**
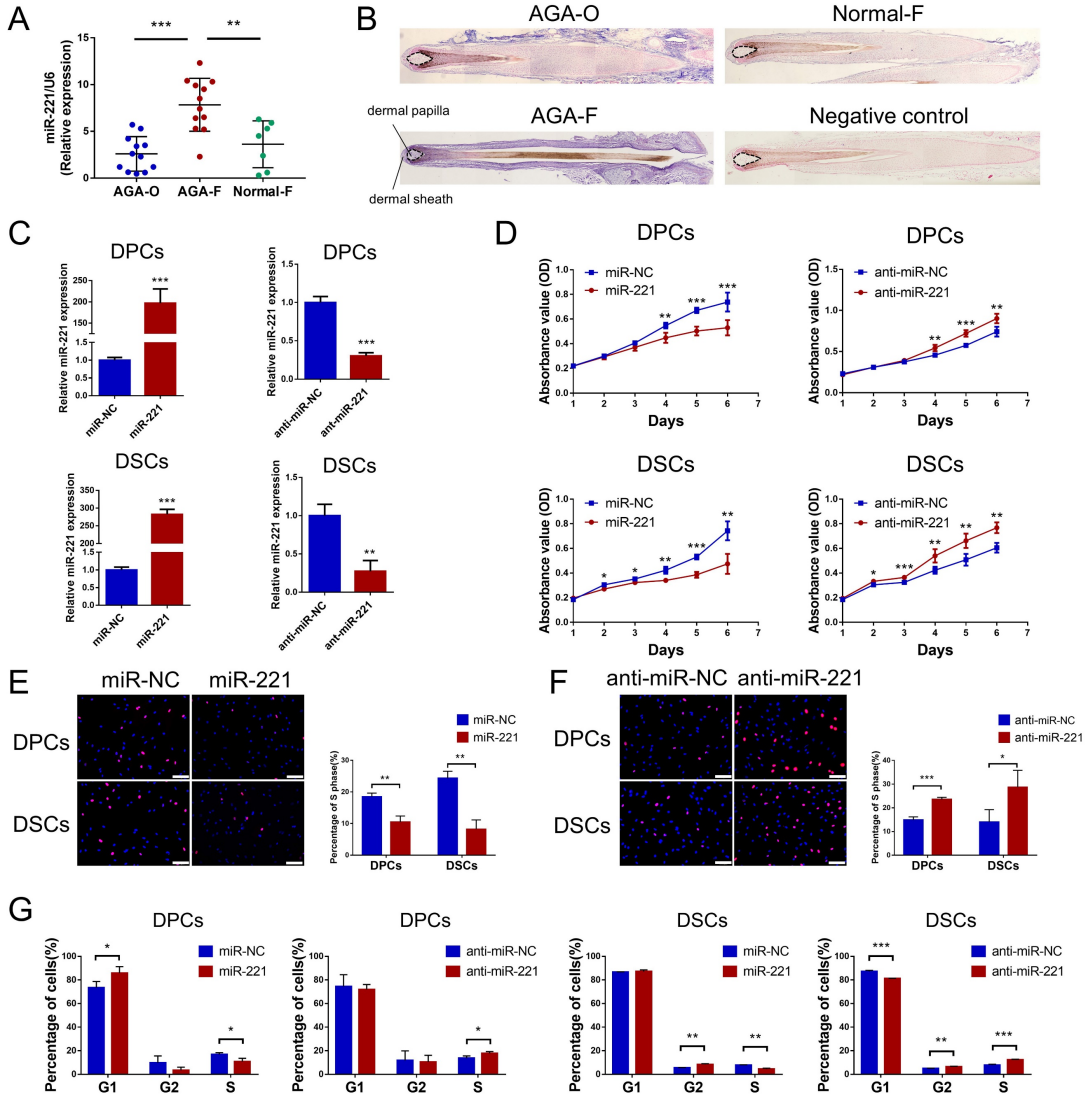
**miR-221 inhibits the proliferation of DPCs and DSCs. (A)** The expression of miR-221 in hair follicle sample of AGA-O (n = 12), AGA-F (n = 12) and Normal-F (n = 7) groups. **(B)** In situ hybridization of miR-221 in AGA-O, AGA-F and Normal-F hair follicles. **(C)** qPCR was performed to detect the mRNA expression of miR-221 in DPCs and DSCs, both transfected with miR-221 angomir or antagomir. CCK-8 assay **(D)**, EdU assay **(E&F)** and cell cycle assay **(G)** of DPCs and DSCs were performed after transfected with miR-221 angomir or antagomir. Student's t-test, mean ± SD, *P < 0.05; **P < 0.01; ***P < 0.001. Scale bars represent 100 μm in **(E&F)**.

Previous analysis of miRNA expression profiles has uncovered miRNAs with elevated expression in balding hair follicles, including miR-221, miR-125b, miR-106a, and miR-410^31^. In our recent miRNA expression profiles (GSE111788), we also confirmed the upregulation of miR-221 in balding hair bulge and hair bulb. To investigate the impact of these miRNAs on DPCs and DSCs proliferation, we identified cultured cells expressing the specific markers ALP and NCAM for DPCs, and α-SMA and NCAM for DSCs, respectively (Fig. S1A). Cell Counting Kit-8 (CCK-8) assay. showed that miR-221 significantly suppressed the proliferative ability of both DPCs and DSCs, while no inhibitory effects were observed for miR-125b, miR-106a, or miR-410 on the proliferation of DPCs and DSCs (Fig. 1D & S2).

Next, quantitative PCR (qPCR) and in situ hybridization experiments were conducted to detect the expression and localization of miR-221 in AGA occipital hair follicle (AGA-O), AGA frontal hair follicle (AGA-F) and frontal hair follicle of the normal subject (Normal-F). Compared with the expression in AGA-O and Normal-F groups, the expression of miR-221 was markedly upregulated in the AGA-O group, particularly in the dermal papilla and dermal sheath compartments within the hair follicle (Fig. 1A&B). To investigate the biological effect of miR-221 on DPCs and DSCs, we transfected the miR-221 angomir (named miR-221 group) and antagomir (named anti-miR-221 group) into DPCs and DSCs, and confirmed the transfection efficiency via qPCR (Fig. 1C). 5-ethynyl-2'-deoxyuridine (EdU) and cell cycle assays were used to further evaluate the role of miR-221 in DPCs/DSCs proliferation and cell cycle regulation. Overexpression of miR-221 significantly suppressed cell growth and G1 to S cell cycle transition in DPCs and DSCs, whereas suppression of miR-221 markedly promoted DPCs/DSCs proliferation and induced G1/S transition (Fig. 1E-G&S4), suggesting miR-221 inhibits DPCs/DSCs proliferation by arresting cells at G1/G0 phase.

### miR-221 restrains hair growth and induces the anagen-catagen transition

**Figure 2:**
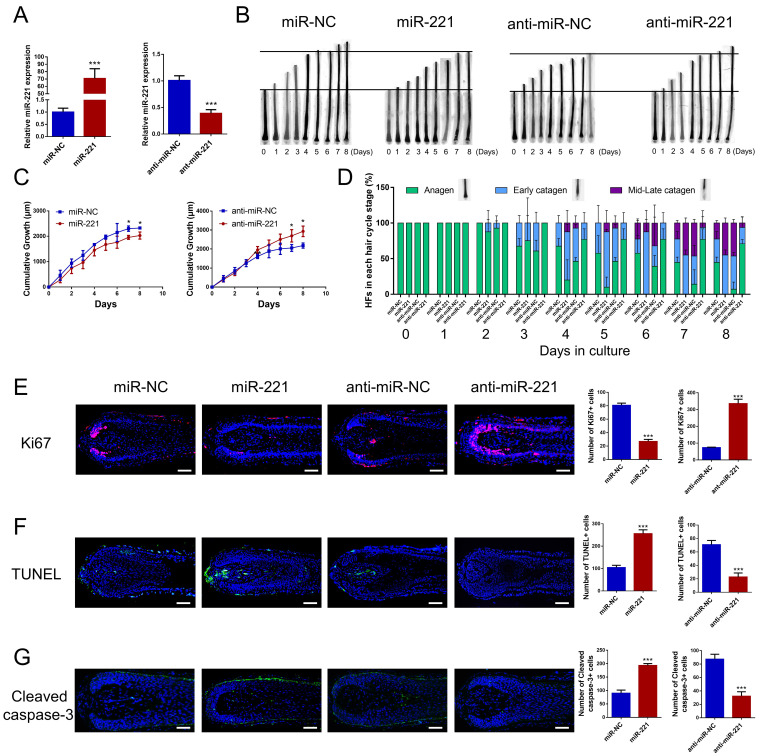
**miR-221 restrains hair follicle growth and induces the anagen-catagen transition in vitro**. **(A)** qPCR was performed to detect the mRNA expression of miR-221 in hair follicles transfected with miR-221 angomir or antagomir. **(B&C)** The growth of hair follicles transfected with miR-221 angomir or antagomir (n = 10). **(D)** The hair cycle stage of hair follicles transfected with miR-221 angomir or antagomir (n = 10). **(E-G)** The expression of Ki67 and cleaved caspase-3 as well as TUNEL staining of hair follicles transfected with miR-221 angomir or antagomir (n = 5). Student's t-test, mean ± SD, *P < 0.05; **P < 0.01; ***P < 0.001. Scale bars represent 100 μm in **(E-G)**.

To investigate the effect of miR-221 on hair growth and hair cycle, we transfected miR-221 angomir and antagomir into AGA hair follicle organ in vitro and confirmed the transfection efficiency using qPCR (Fig. 2A). The cultured hair follicle continued to grow while maintaining normal morphology and structure (Fig. S1B). Overexpression of miR-221 significantly inhibited hair growth and stimulated anagen-catagen transition, while suppression of miR-221 promoted hair growth and lengthened the anagen phase (Fig. 2B-D). To further examine the underlying cellular mechanisms, we performed a quantitative analysis of Ki67, a cellular marker of proliferation, by immunofluorescence staining. Results revealed a significant decrease in Ki67-positive cells in hair follicles treated with miR-221 angomir, and a corresponding increase in Ki67-positive cells in hair follicles Streated with miR-221 antagomir (Fig. 2E). Additionally, we assessed levels of apoptosis using TUNEL and cleaved caspase-3 markers. Strikingly, hair follicles overexpressing miR-221 exhibited a marked increase in TUNEL and cleaved caspase-3 positive cells, indicating increased apoptosis, while hair follicles with miR-221 inhibition showed a marked reduction in TUNEL and cleaved caspase-3 positive cells (Fig. 2F&G).

**Figure 3:**
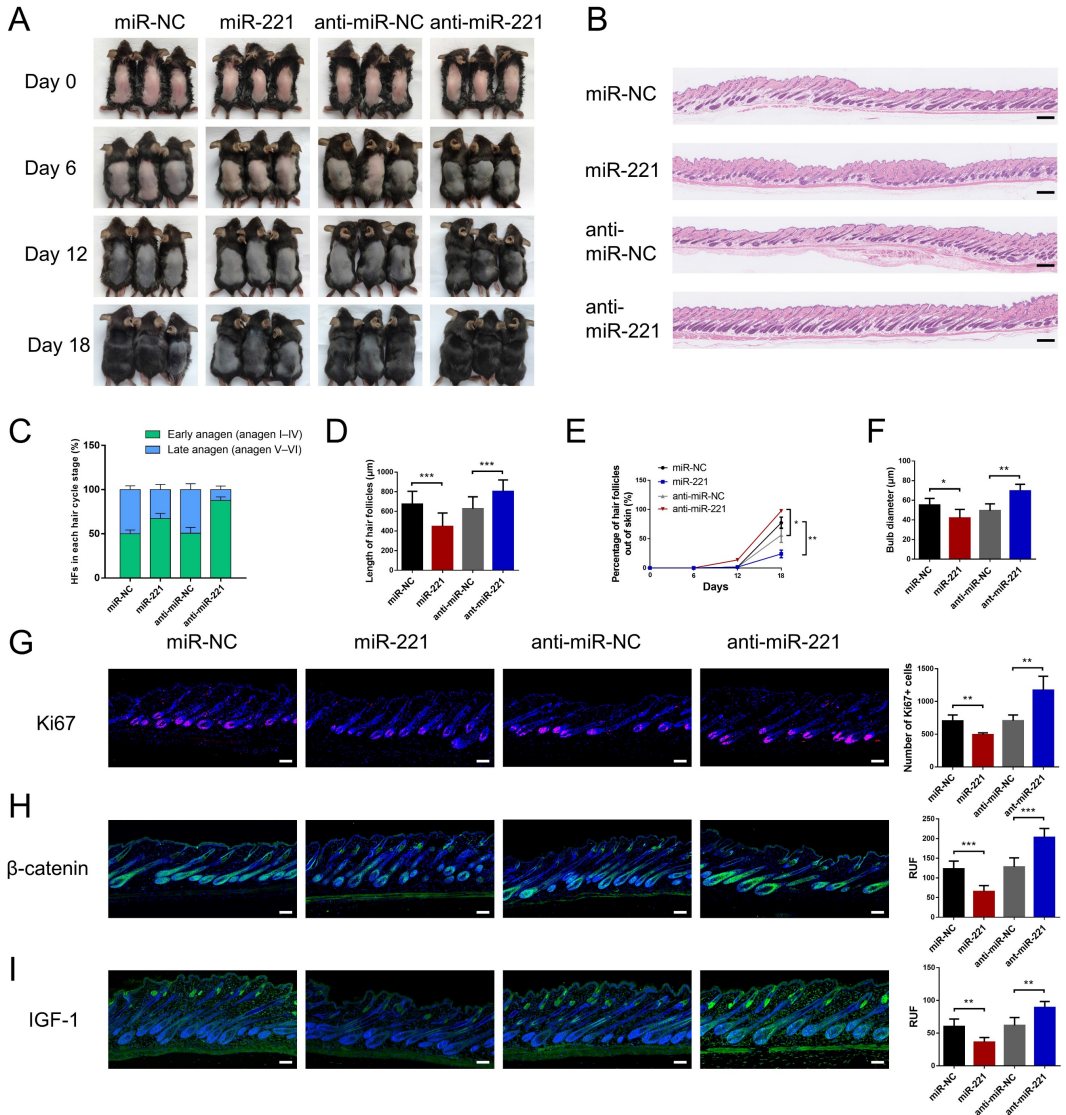
**miR-221 restrains hair follicle growth in vivo**.** (A)** After hair shaving, mice dorsal skin was intradermal injected with miR-221 angomir or antagomir every 5 days. The dorsal skin was photographed on 0, 6, 12, and 18 days. **(B)** On day 18, HE-stained sections of the injection area on the dorsal skin were obtained from each group for analysis. **(C)** The percentage of hair follicle in early anagen and late anagen on day 18 in each group (n = 5).** (D)** The length of hair follicle on day 18 in each group (n = 36), Student's t-test, mean ± SD.** (E)** Areas with hair regrowth out of skin were quantified using Image-Pro Plus software (n = 5).** (F)** The diameter of hair follicle in HE-stained sections of each group (n = 5). **(G-I)** The expression of Ki67, β-catenin and IGF-1 in each group (n = 5). Student's t-test, mean ± SD, *P < 0.05, **P < 0.01, ***P < 0.001. Scale bars represent 100 μm in **(B&G-I)**.

We then intradermally injected the miR-221 angomir and antagomir into shaved dorsal skin of 3-week-old C57BL/6 mice every 5 days, sequential photographs were taken on day 6, 12, and 18 following the first injection. Patches of dorsal skin were fixed for Hematoxylin and Eosin (HE) staining 18 days after the first injection. Injection of miR-221 angomir obviously suppressed hair regrowth and telogen-anagen transition (pink skin turned into dark skin), while miR-221 antagomir accelerated hair regrowth and telogen-anagen transition (Fig. 3A-E). The hair follicle diameter was significantly larger in the anti-miR-221 groups and smaller in the miR-221 group (Fig. 3F). Additionally, the dorsal skins of each group were also stained for proliferation marker Ki67 and hair growth markers β-catenin and IGF-1, the expression of Ki67, β-catenin, and IGF-1 were markedly upregulated in the anti-miR-221 group but downregulated in miR-221 group (Fig. 3G-I). These results demonstrate that miR-221 inhibits hair growth and promotes the anagen-catagen transition.

### DHT suppresses DPCs/DSCs proliferation and hair growth via targeting miR-221

**Figure 4:**
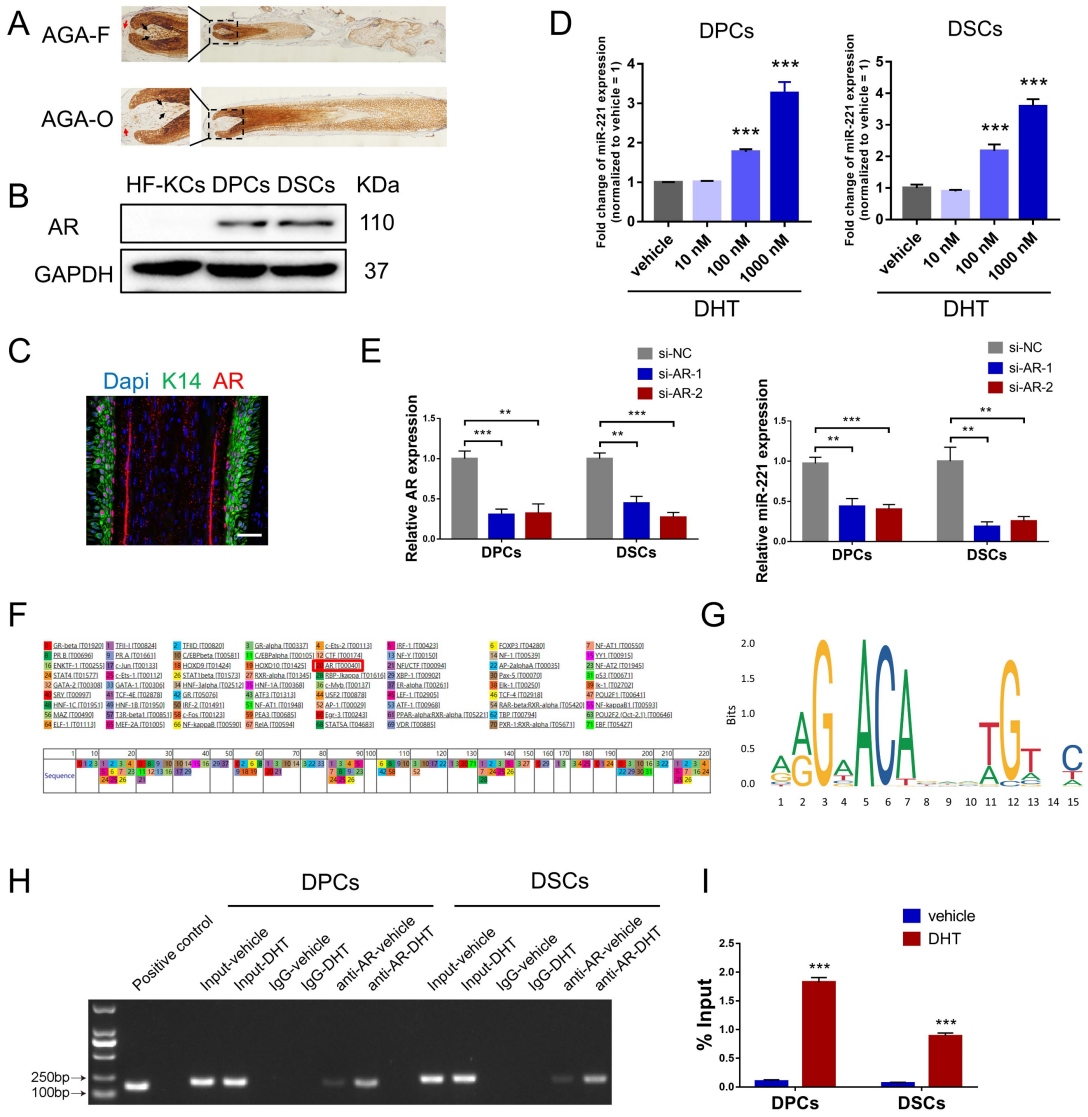
**AR directly regulates the transcription of miR-221 after binding DHT**.** (A)** IHC staining showed the expression of AR in AGA-F and AGA-O hair follicles (black arrow indicated the dermal papilla, red arrow indicated the dermal sheath). **(B)** Western blot experiment showed the expression of AR in DPCs, DSCs and HF-KCs. **(C)** Immunofluorescence experiment showed HF-KCs (K14 positive) expressed AR. **(D)** q-PCR experiment showed the expression of miR-221 in DPCs, DSCs after treatment of DHT at different concentration. Scale bars represent 100 μm. **(E)** q-PCR experiment showed the expression of AR and miR-221 in DPCs and DSCs after treatments of AR siRNAs. **(F&G)** PROMO and JASPAR databases predicted that AR could bind to the promoter region of miR-221. **(H)** PCR gel showing amplification of AR-binding site after ChIP using antibody against AR, DHT could promote the binding of AR to the predicted site in miR-221 promoter region. The gel figures were accompanied by the locations of molecular weight markers. **(I)** ChIP-qPCR showed that DHT could promote the binding of AR to the predicted site in miR-221 promoter region. Student's t-test, mean ± SD, **P < 0.01, ***P < 0.001.

AR is a transcription factor that can be bound and activated by DHT, leading to the expression of genes accountable for the pathogenesis of AGA^32^. Consistent with previous studies^33^,^34^, immunohistochemical staining and western blot experiments demonstrated that AR was predominantly expressed in the dermal papilla and dermal sheath compartments, with higher expression levels observed in balding frontal areas compared to occipital areas (Fig. 4A&B). To investigate the role of DHT in miR-221 regulation, we treated DPCs and DSCs with varying concentrations of DHT (10 nM, 100 nM, and 1000 nM), qPCR analysis revealed a positive correlation between DHT concentration and miR-221 expression (Fig. 4D). Moreover, to determine whether AR regulates miR-221 expression, we used small-interfering RNAs (siRNAs) to knock down AR expression in DPCs and DSCs. qPCR analysis showed a decrease in miR-221 expression after AR knockdown, indicating that AR is an upstream regulator of miR-221 (Fig. 4E).

Intriguingly, both PROMO and JASPAR databases predicted that AR could bind to the promoter region of miR-221, suggesting a potential regulatory role in its transcription (Fig. 4F&G). To investigate this further, we performed chromatin immunoprecipitation (ChIP) assays to assess whether AR was bound to the miR-221 promoter region in DPCs and DSCs. The resulting immunoprecipitated chromatin showed significant enrichment of this specific region compared to negative controls (IgG) and input pulldown, with strengthened enrichments observed after DHT treatment in both cell types (Fig. 4H). Additional ChIP-qPCR analyses confirmed that DHT enhanced AR binding to the promoter region of miR-221 (Fig. 4I).

**Figure 5:**
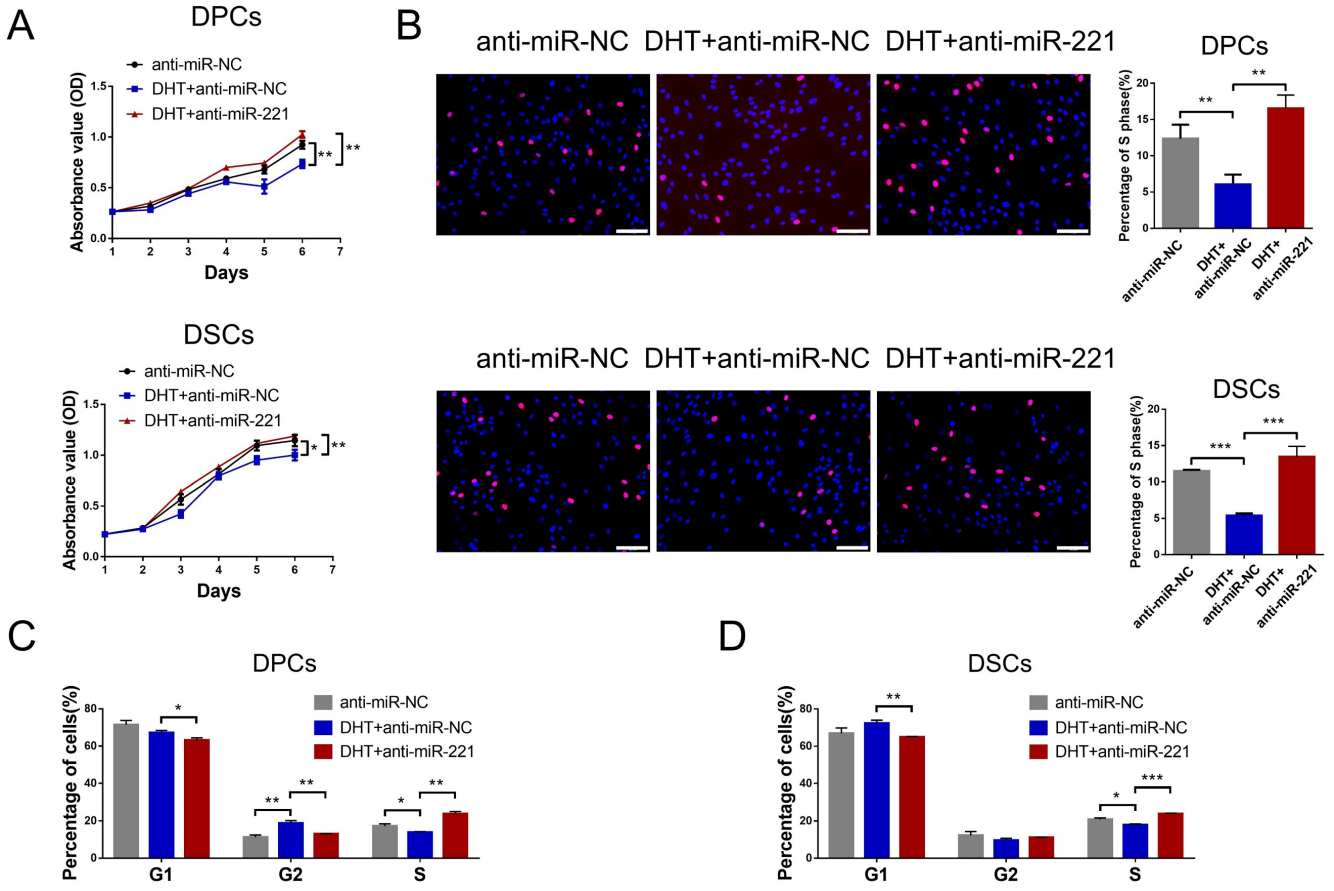
**DHT suppresses DPCs/DSCs proliferation via miR-221**. CCK-8 assay **(A)**, EdU assay **(B)** and cell cycle assay **(C&D)** of DPCs and DSCs were performed after treatments with anti-miR-NC, DHT + anti-miR-NC and DHT + anti-miR-221. Student's t-test, mean ± SD, *P < 0.05; **P < 0.01; ***P < 0.001. Scale bars represent 100 μm in **(B)**.

**Figure 6:**
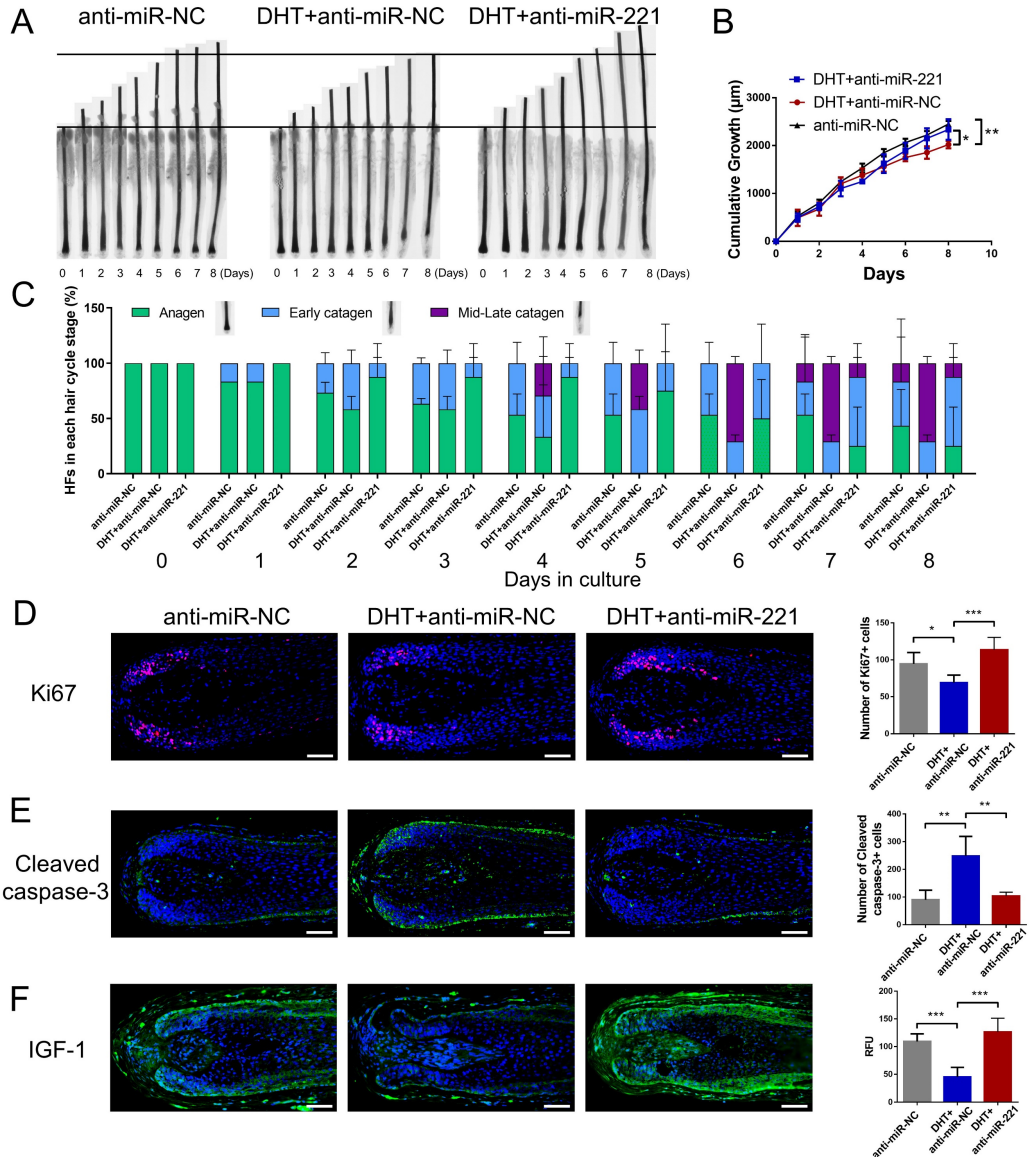
**DHT suppresses hair growth via miR-221**.** (A&B)** The growth of hair follicles treated with anti-miR-NC, DHT + anti-miR-NC and DHT + anti-miR-221 (n = 10). **(C)** The hair cycle stage of hair follicles treated with anti-miR-NC, DHT + anti-miR-NC and DHT + anti-miR-221 (n = 10). **(D-F)** The expression of Ki67, cleaved caspase-3 and IGF-1 in hair follicles treated with anti-miR-NC, DHT + anti-miR-NC and DHT + anti-miR-221 (n = 5), Student's t-test, mean ± SD, *P < 0.05; **P < 0.01; ***P < 0.001. Scale bars represent 100 μm in **(D-F)**.

We next investigated whether DHT regulates cell proliferation and hair growth by targeting miR-221. Using CCK-8, EdU, and cell cycle assays, we found that DHT significantly suppressed DPC and DSC proliferation and G1 to S cell cycle transition. Encouragingly, treatment with a miR-221 antagomir effectively rescued this suppression (Fig. 5A-D&S5). Hair follicle organ culture experiments further revealed that DHT inhibited hair growth and accelerated anagen-catagen transition, while knockdown of miR-221 abrogated the effects of DHT on hair growth and hair cycle (Fig. 6A-C). Immunostaining results further demonstrated that DHT decreased Ki67 and IGF1 expression and increased cleaved caspase-3 expression, whereas the knockdown of miR-221 abolished these effects (Fig. 6D-F). Collectively, these data suggest that DHT regulates hair growth and DPC/DSC proliferation, at least in part, by targeting miR-221.

### miR-221 suppresses DPCs/DSCs proliferation and hair growth via targeting IGF-1

**Figure 7:**
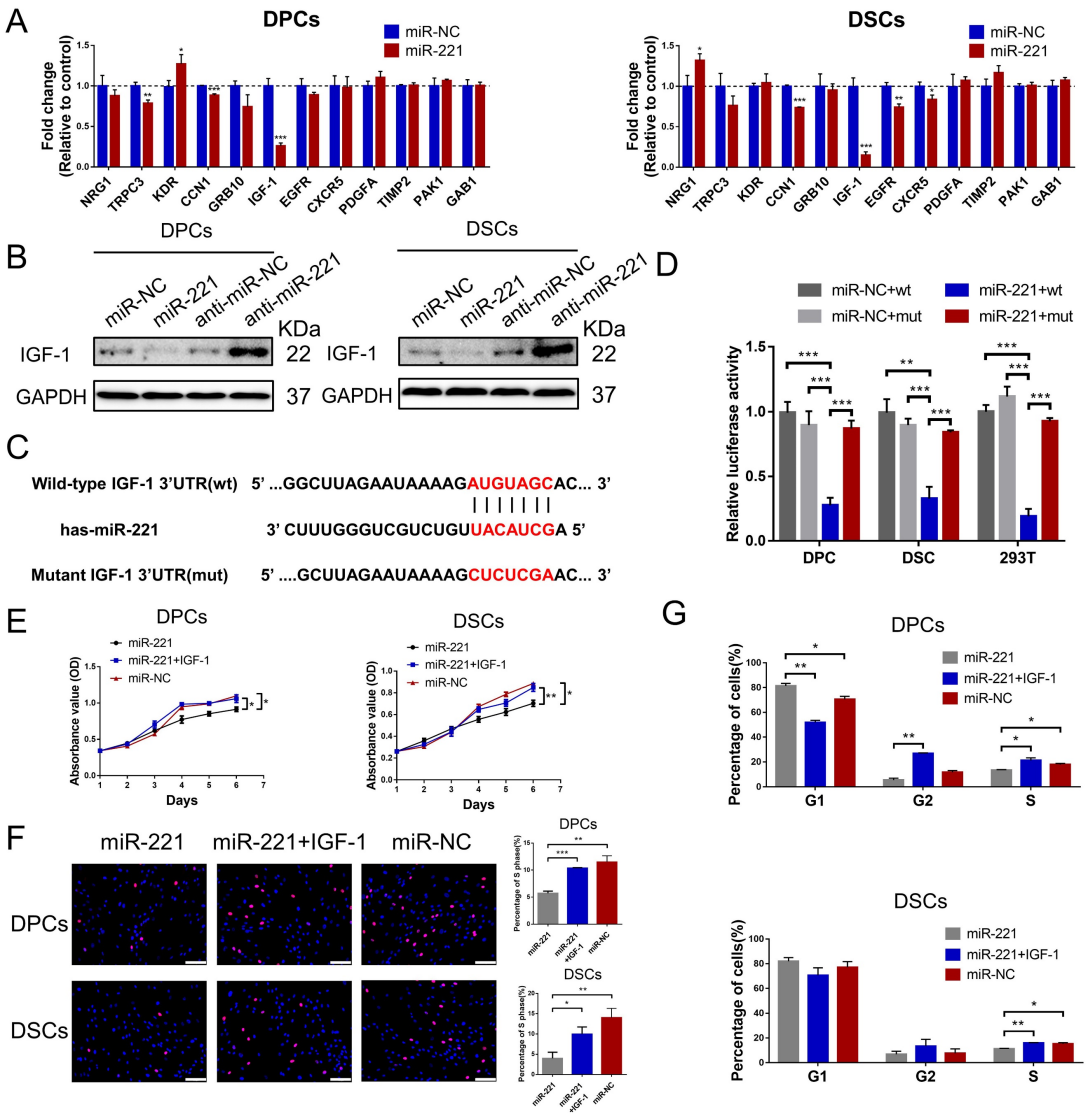
**miR-221 inhibits the proliferation of DPCs and DSCs via targeting IGF-1**.** (A)** qPCR analysis was performed to detect the mRNA expression of candidate genes in DPCs and DSCs transfected with miR-221 angomir.** (B)** Western blot analysis was performed to detect the protein expression of IGF-1 in DPCs and DSCs, both transfected with miR-221 angomir or antagomir.** (C)** miR-221 and its putative binding sequences in the 3'UTR of IGF-1. A mutation was generated in the complementary site that bound to the seed region of miR-221. **(D)** Luciferase reporter assay was used to determine whether miR-221 directly targeted the IGF-1 3'UTR. CCK-8 assay **(E)**, EdU assay **(F)** and cell cycle assay **(G)** of DPCs and DSCs were performed after treatments with miR-221, miR-221 + IGF-1 and miR-NC. Scale bars represent 100 μm in **(F)**. Student's t-test, mean ± SD, *P < 0.05; **P < 0.01; ***P < 0.001.

To identify the potential target gene of miR-221, we performed a bioinformatic analysis using the TargetScan database. 12 candidate genes that have been reported in hair follicle were screened out from the database. Among these genes, IGF-1 exhibited the most significant fold decrease in mRNA expression following miR-221 overexpression (Fig. 7A). Based on these results, we designated IGF-1 as a putative target of miR-221. In Western blot experiments, we further confirmed that IGF-1 protein expression was downregulated after miR-221 overexpression and upregulated following miR-221 inhibition (Fig. 7B).

Dual-luciferase reporter assays were performed to determine whether miR-221 could directly target the 3'UTR region of IGF-1. 3'UTR fragment of IGF-1 containing miR-221 binding site (wt) or its mutant fragments (mut) were cloned into luciferase report vectors psiCHECK-2 (Fig. 7C). The wt or mut 3'UTR vector was co-transfected with miR-221 or negative control into DPCs, DSCs, and 293 T cells. The results showed that miR-221 markedly attenuated the luciferase activity of wide-type IGF-1 3'UTR, whereas the effect was abrogated after the 3'UTR binding site of IGF-1 was mutated (Fig. 7D).

**Figure 8:**
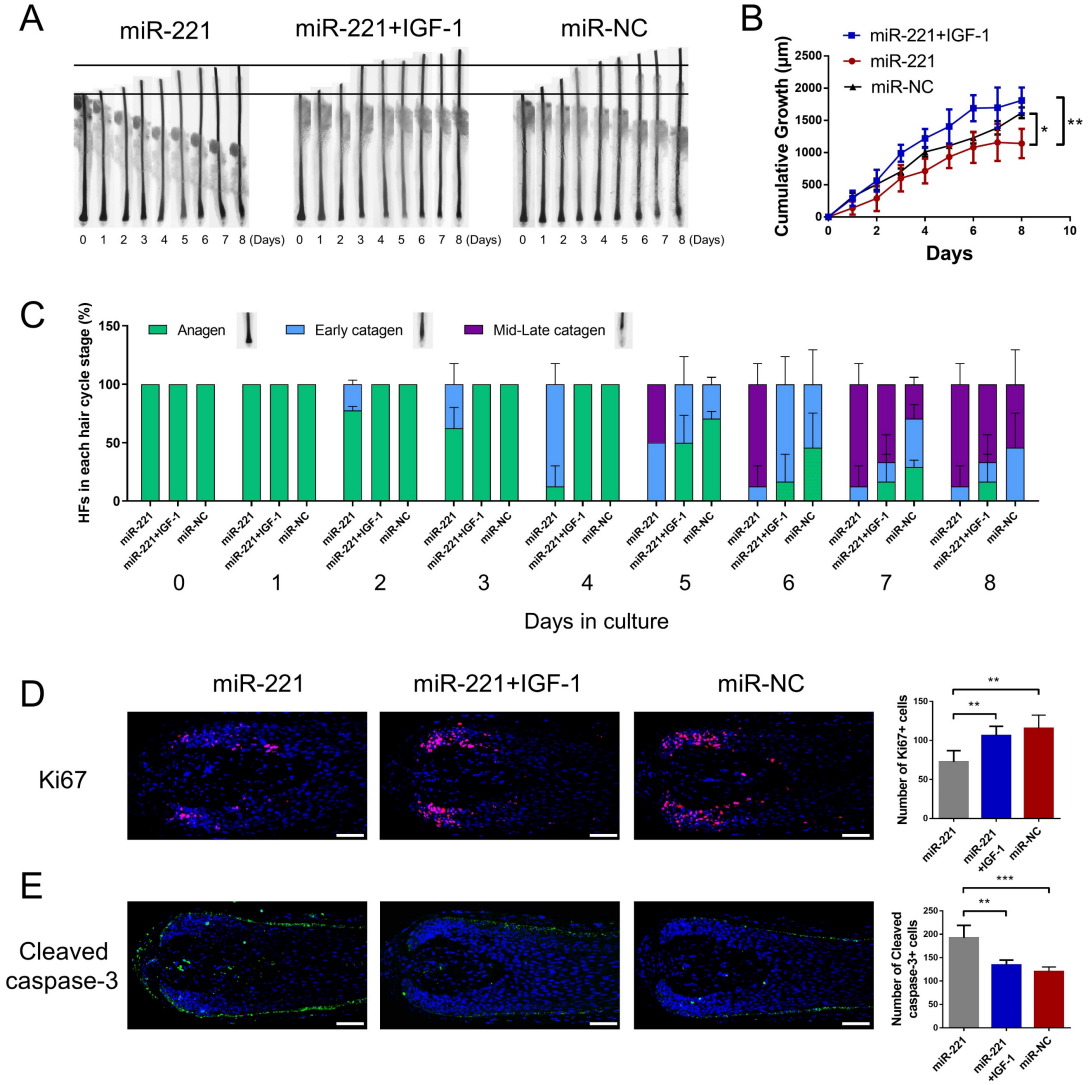
**miR-221 inhibits hair growth via targeting IGF-1**.** (A&B)** The growth of hair follicles treated with miR-221, miR-221 + IGF-1 and miR-NC (n = 10). **(C)** The hair cycle stage of hair follicles treated with miR-221, miR-221 + IGF-1 and miR-NC (n = 10). **(D&E)** The expression of Ki67 and cleaved caspase-3 in hair follicles treated with miR-221, miR-221 + IGF-1 and miR-NC (n = 5). Scale bars represent 100 μm in **(D&E)**. Student's t-test, mean ± SD, *P < 0.05; **P < 0.01; ***P < 0.001.

Subsequently, we investigated the role of IGF-1 in the miR-221-mediated suppression of DPCs/DSCs proliferation and hair growth. CCK-8, EdU, and cell cycle assays showed that IGF-1 was able to restore the miR-221-induced inhibition of cell proliferation and G1 to S cell cycle transition (Fig. 7E-G&S6). Hair follicle organ culture experiments showed that IGF-1 could rescue the miR-221-mediated suppression of hair growth and induction of anagen-catagen transition (Fig. 8A-C). Moreover, immunostaining results showed that IGF-1 abrogated the miR-221-mediated decrease of Ki67 expression and increase of cleaved caspase-3 expression (Fig. 8D&E). Taken together, these results suggest IGF1 plays an important role in the inhibitory effect of miR-221 on DPCs/DSCs proliferation and hair growth.

### miR-221 is an upstream regulator of the MAPK pathway and PI3K/AKT pathway

**Figure 9:**
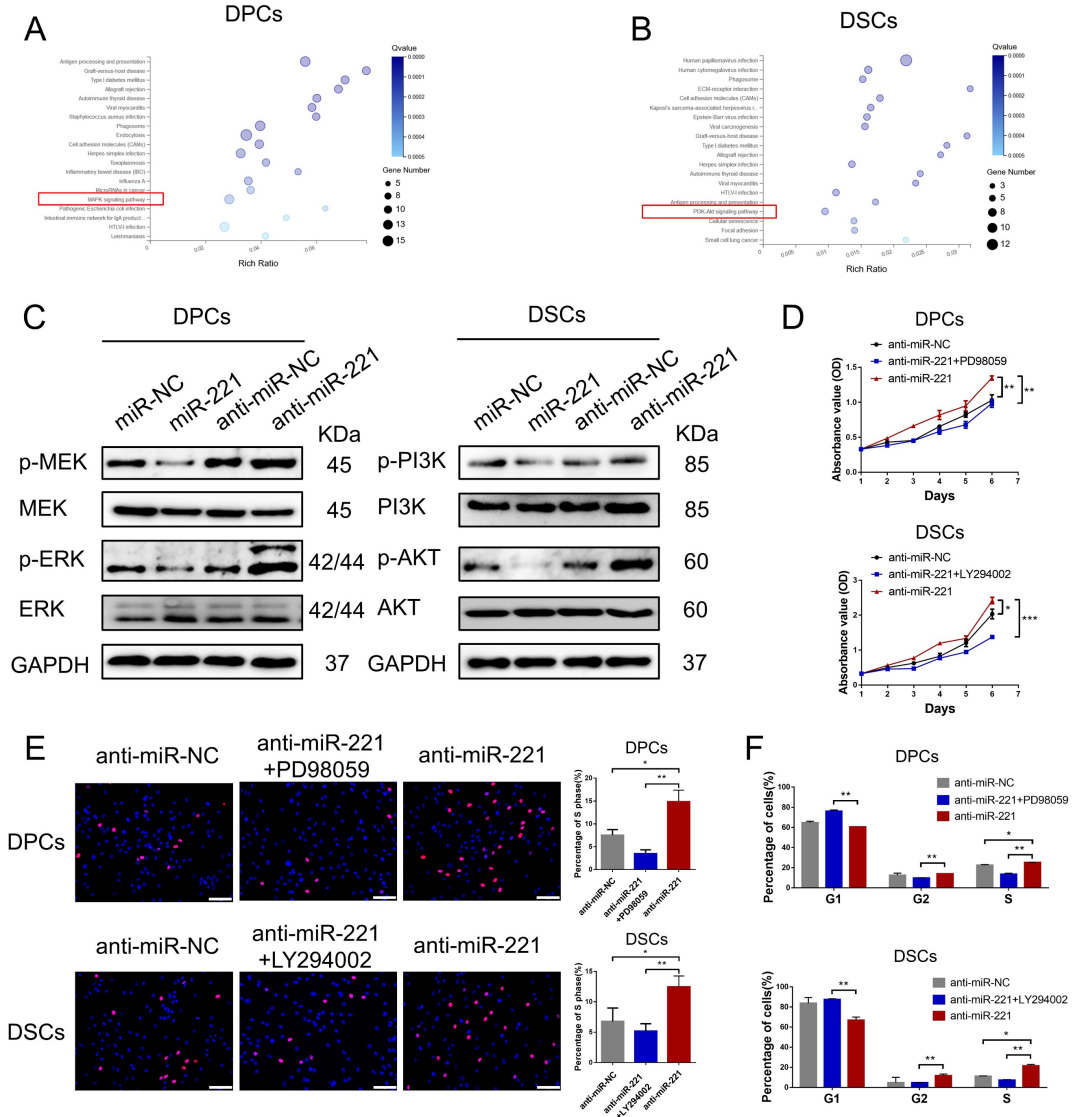
** miR-221 regulate MAPK pathway and PI3K/AKT pathway in DPCs and DSCs respectively. (A&B)** KEGG pathway enrichment analysis of DPCs and DSCs treated with miR-221 angomir or miR-NC. **(C)** Western blot experiments were used to analyze the expression of relevant proteins in MAPK pathway and PI3K/AKT pathway after miR-221 knockdown and overexpression in DPCs and DSCs respectively. CCK-8 assay **(D)**, EdU assay **(E)** and cell cycle assay **(F)** were performed after DPCs treated with anti-miR-NC, anti-miR-221 + PD98059 and anti-miR-221, as well as DSCs treated with anti-miR-NC, anti-miR-221 + LY294002 and anti-miR-221. Scale bars represent 100 μm in **(E)**. Student's t-test, mean ± SD, *P < 0.05; **P < 0.01; ***P < 0.001.

To shed light on the mechanisms underlying the regulatory effects of miR-221 on DPCs/DSCs proliferation, we performed RNA sequencing (RNA-seq) analysis on both miR-221-overexpressing and control DPCs/DSCs (GSE205075). By conducting Kyoto Encyclopedia of Genes and Genomes (KEGG) pathway enrichment analysis, we found that differential gene expression (fold change >2) in DPCs was mainly associated with antigen processing and presentation, graft-versus-host disease, miRNA in cancer, and the MAPK signaling pathway, among others (Fig. 9A). Similarly, differential gene expression (fold change >2) in DSCs was mainly associated with human papillomavirus infection, phagosome, ECM-receptor interaction, and the PI3K/AKT signaling pathway, etc. (Fig. 9B).

Subsequent western blot experiments revealed that overexpression of miR-221 led to downregulation of phosphorylated MEK and ERK levels in DPCs, while knockout of miR-221 resulted in their upregulation, with no significant changes observed in the total protein amount of MEK and ERK. Similarly, overexpression of miR-221 led to decreased phosphorylation levels of PI3K and Akt in DSCs, whereas inhibition of miR-221 resulted in their upregulation, with no significant changes observed in the total protein amount of PI3K and Akt (Fig. 9C). Furthermore, we found that inhibitors of the MAPK pathway (PD98059) and the PI3K/ATK pathway (LY294002) effectively neutralized the promotion effect of miR-221 antagomir on proliferation and G1 to S cell cycle transition in DPCs and DSCs, respectively, as demonstrated by CCK-8, EdU, and cell cycle assays (Fig. 9D-F&S7). Altogether these findings suggest that the MAPK pathway in DPCs and PI3K/AKT pathway participates in the inhibition effect of miR-221 on DPC and DSCs proliferation.

### miR-221 regulates MAPK pathway and PI3K/AKT pathway via targeting IGF-1

**Figure 10:**
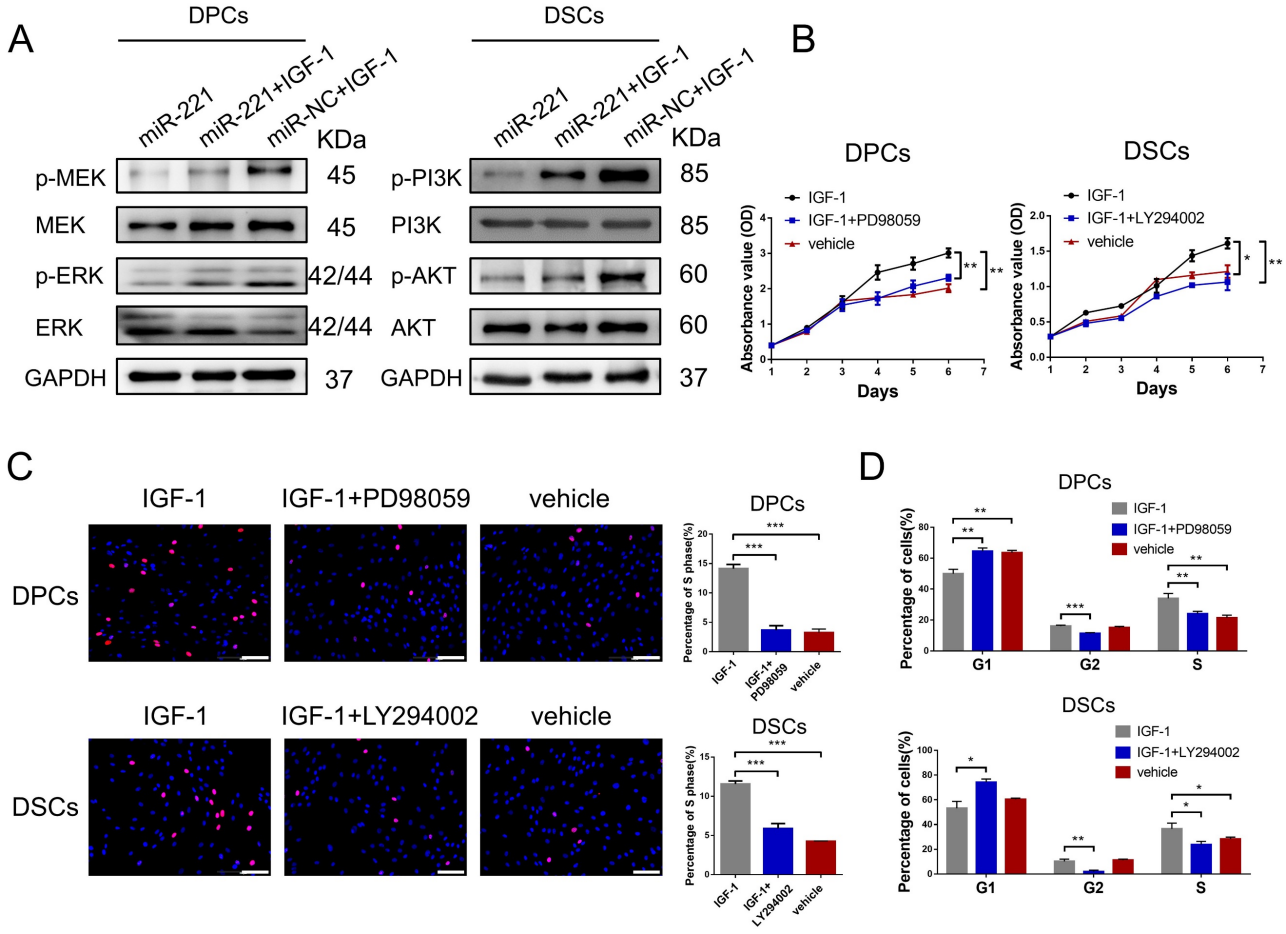
**miR-221 regulate MAPK pathway and PI3K/AKT pathway via targeting IGF-1**.** (A)** Western blot experiments were used to analyze the expression of relevant proteins in MAPK pathway and PI3K/AKT pathway in DPCs and DSCs respectively after treatments of miR-221, miR-221 + IGF-1 and miR-NC + IGF-1. CCK-8 assay **(B)**, EdU assay **(C)** and cell cycle assay **(D)** were performed after DPCs treated with IGF-1, IGF-1 + PD98059 and vehicle, as well as DSCs treated with IGF-1, IGF-1 + LY294002 and vehicle, Student's t-test, mean ± SD, *P < 0.05; **P < 0.01; ***P < 0.001. Scale bars represent 100 μm in **(C)**.

To investigate the role of IGF-1 in the regulatory effects of miR-221 on MAPK and PI3K/AKT pathways, we conducted western blot experiments to detect changes in these pathways in DPCs/DSCs following treatment with miR-221, miR-221+IGF-1, or IGF-1 alone. Remarkably, our results demonstrated that IGF-1 was able to rescue the miR-221-mediated suppression of phosphorylation levels of p-MEK and p-ERK in DPCs, as well as the phosphorylation levels of p-PI3K and p-Akt in DSCs (Fig. 10A).

Next, CCK-8, EdU, and cell cycle assays were conducted to investigate the biological function of IGF-1 in DPCs and DSCs. Significantly, IGF-1 promotes cells proliferation and G1 to S cell cycle transition in both DPCs and DSCs. Additionally, the promotion effects were effectively abolished by the inhibitors of the MAPK and PI3K/AKT pathways, PD98059 and LY294002, respectively (Fig. 10B-D&S8). Collectively, our results suggest that IGF-1 stimulates DPCs and DSCs proliferation and G1 to S cell cycle transition via activating the MAPK and PI3K/AKT pathways, respectively.

**Figure 11:**
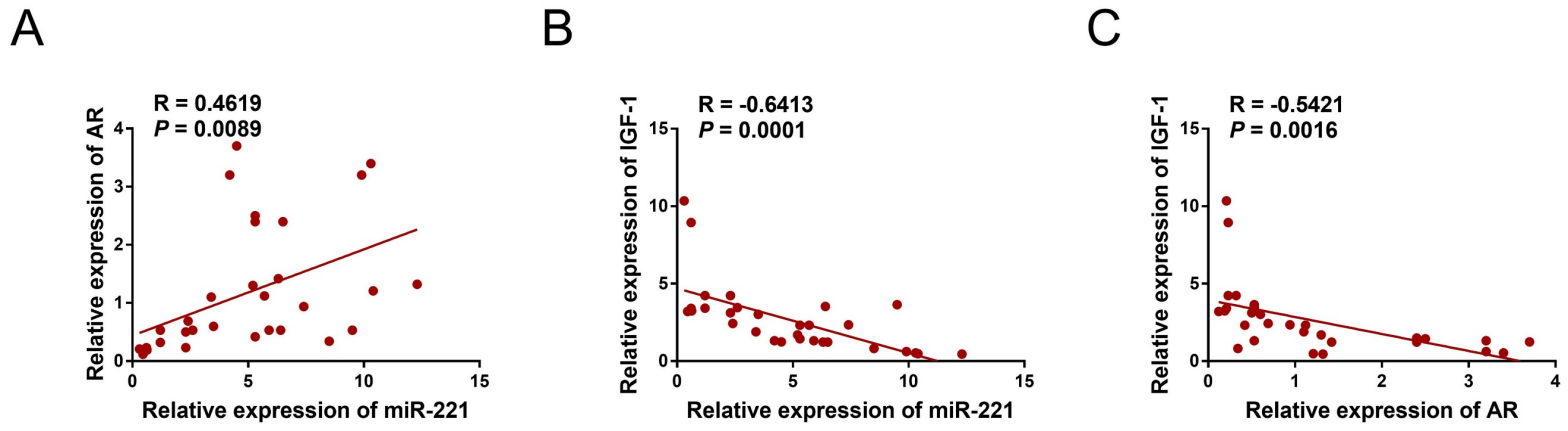
** Correlation analysis in hair follicles. (A)** qPCR assay of hair follicle samples was performed to analyze the mRNA expression correlation between miR-221 and AR (n =31). **(B)** qPCR assay of hair follicle samples was performed to analyze the mRNA expression correlation between miR-221 and IGF-1 (n =31).** (C)** qPCR assay of hair follicle samples was performed to analyze the mRNA expression correlation between AR and IGF-1 (n =31).

We collected a total of 12 samples of AGA-O hair follicles, 12 samples of AGA-F hair follicles, and 7 samples of Normal-F hair follicles to investigate the mRNA expression levels of AR, miR-221, and IGF-1 using qPCR experiments. Interestingly, our findings revealed a negative correlation between the expression of IGF-1 and that of miR-221 and AR, whereas the expression of miR-221 was positively correlated with that of AR (Fig. 11A-C), indicating the existence of an AR/miR-221/IGF-1 axis that modulates DPCs/DSCs proliferation and hair growth.

## Discussion

Aberrant AR function has been detected in various diseases, including AGA, cancer, hypogonadism, androgen insensitivity syndrome, muscle atrophy, and osteoporosis^35^-^37^. In these conditions, AR that is not bound to a ligand is retained in the cytoplasm, but upon binding with dihydrotestosterone (DHT), a potent androgenic hormone, it translocates into the nucleus and promotes the transcription of targeted genes^38^. Therefore, it is crucial to comprehend the underlying molecular mechanisms that regulate AR activity in these disease states. Specifically concerning AR-related diseases, the role of AR and its interaction with miRNAs is inadequately described and poorly explored. Recent studies have shed new light on the interaction between AR and miRNAs in various diseases, including prostate cancer, ovarian cancer, and breast cancer, among others^39^. AR can directly regulate miRNAs by binding to specific DNA sequences known as androgen response elements (AREs) in their target gene promoters, resulting in either transcriptional activation or repression, albeit in rare cases. For example, in prostate cancer, AR can negatively regulate the expression of β-catenin by enhancing miR-4496 expression through direct binding to the AREs in the promoter of miR-4496^40^. Similarly, in breast cancer, treatment of cells with DHT results in up to a 13-fold increase in the expression of miR-328-3p due to AR binding to the promoter of miR-328-3p^35^. In this study, we have identified miR-221 as a target gene of AR that plays a significant role in the suppression of proliferation in DPCs/DSCs and hair growth mediated by DHT/AR. However, we acknowledge that other miRNAs may also be regulated by AR in DPCs/DSCs, and future research should aim to explore more miRNAs downstream of AR and establish a regulatory network of miRNAs involved in the pathogenesis of AGA. Obtaining a deeper understanding of AR-miRNA interactions may lead to the development of improved diagnostic tools and provide new therapeutic approaches for this disorder.

In situ hybridization experiments have shown that miR-221 is highly expressed not only in the dermal papilla and dermal sheath region but also in the epithelial component of the hair follicle (Fig. 1B). The epithelial component is composed of various lineages of keratinocytes and their progenitor/stem cells^41^. There exists a closed signaling interaction between the dermal and epithelial compartments of the hair follicle^42^. To investigate the effects of miR-221 on the biological function of hair follicle keratinocytes (HF-KCs), we performed western blot assays and found that overexpression of miR-221 significantly reduced IGF-1 expression, leading to a subsequent decline in the proliferation ability of HF-KCs. In contrast, knockout of miR-221 results in elevated IGF-1 levels and enhances the proliferation of HF-KCs. Moreover, exogenous IGF-1 counteracts the inhibitory effect of miR-221 on the proliferation of HF-KCs (Fig. S3). These results emphasize the pivotal role played by the miR-221/IGF-1 pathway in regulating the proliferation of HF-KCs. Therefore, the miniaturization of hair follicles induced by miR-221 may involve two distinct mechanisms. Firstly, miR-221 acts directly on the hair follicle keratinocytes and inhibits their proliferation. Secondly, miR-221 targets dermal papilla cells, leading to reduced secretion of growth signals that indirectly inhibit hair follicle proliferation. Our findings provide important insights into the complex regulatory network behind the development of AGA.

DHT is the most potent hormone among androgens. Following conversion from testosterone by the 5-alpha reductase enzyme, DHT binds to AR in hair follicles and stimulates the transcription of genes responsible for AGA^43^. In our study, immunostaining for AR showed that AR was expressed in the dermal papilla, dermal sheath, and outer root sheath (ORS) area (comprised of HF-KCs), co-localization analysis of K14 (HF-KCs marker) and AR signifies the expression of AR in HF-KCs. However, western blot experiments indicated that AR was predominantly expressed in the dermal papilla and dermal sheath, rather than the ORS area (Fig. 4A-C). The expression of AR in the HF-KCs has long been debated, Asada et al found that AR mRNA was expressed not only in the dermal papilla and dermal sheath compartments but also in the ORS regions of human scalp hair follicles^44^. Kretzschmar et al reported that AR was expressed in the ORS and played a key role in epidermal stem cell fate selection in mouse hair follicles^45^. However, Thornton et al revealed that AR expression was not observed in the ORS of human scalp hair follicles^46^. Additional experiments are required to determine the expression and function of AR in HF-KCs to further understand the etiology and pathogenesis of AGA.

In conclusion, as summarized in our working model in **Scheme 1**, upon binding with DHT, AR translocates to the nucleus and directly triggers the transcription of miR-221. Subsequently, miR-221 inhibits the MAPK pathway in DPCs and the PI3K/AKT pathway in DSCs via targeting IGF-1. This leads to the suppression of DPCs and DSCs proliferation, ultimately resulting in hair loss. Thus, we have uncovered a novel AR/miR-221/IGF-1 pathway that provides a mechanistic explanation for the androgen-mediated pathogenesis of AGA. Our study suggests that miR-221 might serve as a potential biomarker and/or therapeutic target for AGA progression.

## Methods

### Patients and samples

A total of 23 male patients with androgenetic alopecia (AGA), with a Hamilton-Norwood baldness scale score of 3-5 and an age range of 23-46 (median age 34.2), were recruited for this study, along with 5 healthy male controls (age range of 22-38, median age of 27.5). Patients diagnosed with systemic diseases were excluded from the study. Scalp hair follicles were obtained from patients undergoing hair transplantation surgery using follicular unit extraction (FUE) techniques. Approximately 30-50 hair follicles were collected from each participant in a specified area. Specifically, in AGA patients, 30-50 hair follicles were harvested from both the frontal balding area (AGA-F) and occipital non-balding area (AGA-O), respectively, while in healthy male controls, 30-50 hair follicles were collected from the frontal non-balding area (Normal-F). All study participants provided written consent, and the study protocol was approved by the research ethics board at Nanfang Hospital, Southern Medical University, Guangzhou, China, in accordance with the principles of the Declaration of Helsinki.

### Immunohistochemistry (IHC), immunofluorescence, and TUNEL staining

The Dako Envision two-step method was employed for immunohistochemistry as per the manufacturer's guidelines^47^. Deparaffinization and hydration were carried out by sequential treatment with graded alcohol and water for the hair follicle or dorsal skin sections (3μm). Antigen retrieval was performed by high-pressure heat in citrate buffer for 5 minutes.

For IHC, endogenous peroxidase activity was eliminated by treating the sections with 3% H2O2 for 15 minutes, following which AR (1:500, Abcam, London, UK) was incubated overnight at 4℃. The sections were washed thrice with PBS before being incubated with biotinylated secondary antibodies (1:300, Dako, Denmark; Glostrup) at room temperature for 60 minutes. Sections were then observed using 3,3'-iaminobenzidine DAB and counterstained with hematoxylin for 30 seconds, followed by dehydration and mounting in neutral gum.

For immunofluorescence, permeabilization was achieved by treatment with 0.5% Triton X-100 in PBS, followed by blocking with 0.5% BSA in PBS for 1 hour. Primary antibodies including Ki67 (1:200, Abcam, London, UK), Cleaved caspase-3 (1:1000, CST, Danvers, MA), β-catenin (1:200, CST, Danvers, MA), IGF-1 (1:250, Abcam, London, UK), AR (1:250, Abcam, London, UK), K14 (1:100, Proteintech, Chicago, USA) were incubated overnight at 4℃. After thorough rinsing with PBS, samples were incubated with Alexa Fluor-488 conjugated anti-rabbit secondary antibody (1:200, Abcam, London, UK) for 1 hour at room temperature. Nuclei were counterstained with 4'-6-Diamidino-2-phenylindole (DAPI).

Apoptotic cells were detected using an In Situ Cell Death Detection Kit (Roche, Basel, Switzerland) and TUNEL staining, and nuclei were counterstained with DAPI. Imaging was performed by an Olympus BX63 microscope (Tokyo, Japan), and the images were analyzed using cellSens software (Olympus, Tokyo, Japan). Immunostaining intensity was assessed quantitatively by immunohistomorphometry using ImageJ software (National Institutes of Health, Bethesda, MD), as described previously^48^.

### Microarray

For microarray, all the cells are collected. RNA was prepared (see Supplementary materials for detail). The microarray data reported here have been submitted to the GEO (GSE205075).

### Luciferase reporter assay

The potential direct regulation of IGF-1 by miR-221 was investigated via TargetScan software. A 500-bp fragment of the IGF-1 3'UTR, amplified by PCR primers (designated as wt), was cloned into psiCHECK-2 vectors. GeneTailor Site-Directed Mutagenesis System (Invitrogen) was used to perform site-directed mutagenesis of the miR-221 binding site in the IGF-1 3'UTR (designated as mut). Details of the sequence for wt and mut constructs can be found in **Supplementary Table 2**. To perform reporter assays, cotransfection of the wt or mut vector, and the control vector psiCHECK-2 vector was carried out using miR-221 mimics or control in DPCs, DSCs, and 293T cells. Luciferase activity was measured at 48 h after transfection utilizing the Dual-Luciferase Reporter Assay System (Promega Corporation, Madison, WI, USA).

### Chromatin immunoprecipitation (ChIP) and ChIP-qPCR assay

Chromatin immunoprecipitation (ChIP) assays were conducted in accordance with manufacturer's instructions using a SimpleChIP® Enzymatic Chromatin IP Kit (#9005S, CST, Danvers, MA) to ascertain whether AR was capable of binding to the miR-221 promoter. Firstly, DPCs and DSCs were fixed using 1% formaldehyde to covalently crosslink proteins and DNA prior to chromatin isolation from cells. The resulting crosslinked DNA was then sheared using sonication, generating fragments 200-1,000 base pairs in length, which were subsequently subjected to immunoselection employing protein G magnetic beads, with anti-AR antibody (CST, Danvers, MA) used as a component of the process. Finally, PCR and qPCR were utilized to evaluate enrichment of DNA fragments based on specific primers designed for the putative AR-binding sites within the miR-221 promoter region, using the following primers: 5'-TCTGGCTACTGGGTCTCTGA-3' (forward) and 5'-GCTGATAATGTTGGACTTAACACCC-3' (reverse).

### Statistical analysis

Data were analyzed using SPSS version 19.0 software (SPSS; Chicago, USA). Statistical significance of difference between groups was determined by a two-tailed paired Student's t test. Associations between miR-221 and AR or 221 and IGF-1 gene or AR and IGF-1 were analyzed using Spearman's correlation coefficient. Statistical significance was established at *P* < 0.05.

## Supplementary Material

Supplementary materials and methods, figures and tables.

## Abbreviations

AGA: androgenetic alopecia
DHT: dihydrotestosterone
AR: androgen receptor
DPCs: dermal papilla cells
DSCs: dermal sheath cells
miRNAs: microRNAs
IGF-1: insulin-like growth factor 1
qPCR: quantitative PCR
AGA-O: AGA occipital hair follicle
AGA-F: AGA frontal hair follicle
Normal-F: frontal hair follicle of normal subject
siRNA: small-interfering RNAs
HE: Hematoxylin and Eosin
ChIP: chromatin immunoprecipitation
ORS: outer root sheath
HF-KCs: hair follicle keratinocytes
IHC: immunohistochemistry
