# Supplementary material for: The AR/miR-221/IGF-1 pathway mediates the pathogenesis of androgenetic alopecia
Source: Int J Biol Sci. 2023 Jun 26;19(11):3307–23. doi: 10.7150/ijbs.80481 (PMC10367565; doi:10.7150/ijbs.80481)
Supplement: Supplementary file 1 — Supplementary materials and methods, figures and tables. [file ijbsv19p3307s1.pdf]

Supplementary Materials and Methods

Cell culture and treatment

DPCs, DSCs and HF-KCs were isolated from hair follicles affected by AGA using techniques described previously[1-3]. DPCs and DSCs were cultured in DMEM medium (Gibco, Grand Island, NY, USA) supplemented with 10% fetal bovine serum (FBS) (Gibco, Grand Island, NY, USA) and 100 U/ml penicillin-streptomycin (ScienCell, San Diego, CA. USA) under standard conditions of a humidified incubator with 5% CO2 at 37°C. HF-KCs were cultured in defined keratinocyteserum-free medium (K-SFM, Gibco) supplemented with 100 U/ml penicillin-streptomycin (ScienCell, San Diego, CA. USA) under standard conditions of a humidified incubator with 5% CO2 at 37°C.

For inhibitor treatment, LY294002, a 5 μM PI3K inhibitor (CST, Danvers, MA), or PD98059, a 10 μM MAPK inhibitor (Abcam, London, UK), was added to cultured cells every two days. For treatment with IGF-1 or DHT, 100 ng/mL IGF-1 (Abcam, London, UK) or 1000 nM DHT (Solarbio, Beijing, China) was added to culturing cells every two days. For transfection, angomirs and antagomirs of miR-221, miR-106a, miR-125b, miR-410, as well as AR-specific siRNAs (si-AR-1, si-AR-2), were purchased from GenePharma (Shanghai, China). Cells were plated in six-well plates at a density of 0.5 × 105 cells/mL during exponential growth phase for 24 hours before being transfected with 50 μM miRNAs or siRNAs using Lipofectamine 2000 reagent (Invitrogen; Carlsbad, Calif, USA) in reduced serum medium (OPTI-MEM-I; Invitrogen) according to the manufacturer's instructions.

RNA isolation, reverse transcription, and quantitative real-time PCR

Total RNA was extracted using Trizol reagent (Invitrogen) in accordance with the

manufacturer's protocol. To quantify mRNA expression, polyadenylated total RNA underwent reverse transcription using PrimeScript™ RT Master Mix (TaKaRa, Dalian, China). An SYBR® Premix Ex TaqTM II kit (TaKaRa, Dalian, China) was utilized for real-time PCR on a Roche LightCycler480 system. To quantify miR-221 expression, polyadenylated total RNA underwent reverse transcription using an NCode miRNA First-Strand cDNA Synthesis kit (Invitrogen). Real-time PCR was conducted using an SYBR Green PCR master mix (Applied Biosystems; Foster City, Calif, USA) on a Roche LightCycler480 system. GAPDH or U6 snRNA were used as internal controls. All samples were normalized to these controls, and fold changes were calculated through relative quantification (2-ΔΔCT) as is standard. The specific primers used are detailed and can be found in **Supplementary Table 1**.

Organ culture and treatment

Anagen hair follicles were cultured and measured as previously described[4]. Briefly, the hair follicles were cultured in Williams E medium (Sigma-Aldrich, St. Louis, MO, USA) supplemented with 10 ng/ml hydrocortisone, 2 mM L-glutamine, 10 mg/ml insulin, and 100 U/ml streptomycin for 7 days in 24-well dishes. The hair follicles were photographed using a Leitz Labovert FS inverted microscope (Wetzlar, Germany) and the length was measured every 24 hours for 8 days. The stage of hair cycle was classified according to a previous study[5].

For IGF-1 or DHT treatment, 1000 ng/mL IGF-1 (Abcam, London, UK) or 8000 nM DHT (Solarbio, Beijing, China) was added to the cultured cells every two days. For transfection, hair follicles were transfected with 200 μM miRNAs using Lipofectamine 2000 reagent (Invitrogen; Carlsbad, Calif, USA) according to the manufacturer's protocol.

In vivo experiment

All animals used in this study were purchased from the Experimental Animal Centre at Southern Medical University (Guangzhou, China). After shaving, 3-week-old littermate C57BL/6 mice received intradermal injections of miR-221 angomir, antagomir, angomir control, or antagomir control (3mg/kg) every 5 days, as previously described[6-8]. we used a 3*2 grid pattern to inject at 6 points on the dorsal skin of mice, with a volume of 100μl per injection point. The animals were examined and photographed every 6 days and sacrificed on day 18 for histology and immunofluorescence analysis.

The dorsal skin of the sacrificed mice was excised and fixed in 4% paraformaldehyde at 4 °C for a maximum of 1 week. For histological analysis, the dorsal skin samples were embedded in paraffin blocks and 3 μm thick sections were prepared according to longitudinal sections of hair follicles, followed by hematoxylin and eosin (HE) staining. Digital photomicrographs of representative areas were obtained. Hair bulb diameter was determined using Image-Pro Plus software.

Cell proliferation assays

Cell proliferation assays were conducted using the Cell Counting Kit 8 (CCK-8) (Dojindo; Kumamoto, Japan). Cells were seeded at a density of 1×10^4 cells per well in 96-well plates and cultured in the growth medium. At the indicated time points, the number of cells was measured in triplicate wells by detecting the absorbance at 450 nm.

miRNA in situ hybridization

In situ hybridization assays for mature hsa-miR-221 in hair follicle tissue sections were conducted using digoxin-labeled oligonucleotide probes. The specific hsa-miR-221 probe (5'-GAAACCCAGCAGACAATGTAGCT-3') and negative control scrambled-miR probe

(5'-TTCACAATGCGTTATCGGATGT-3') (Sangon Biotech, Shanghai, China) were utilized. The miRNA in situ hybridization was performed in accordance with previously reported procedures[9]. Optical signals were visualized using an Olympus BX63 microscope (Tokyo, Japan).

Cell cycle analysis and EdU incorporation assay

Cells (1×10^6) were collected and washed with phosphate-buffered saline (PBS). The cells were then resuspended in 1 mL of DNA staining solution (MULTI SCIENCE; Hangzhou, China) and vortexed for 10 seconds. Next, the mixture was incubated at room temperature in the dark for 30 minutes. Flow cytometry was employed to analyze the cell cycle. The proliferating DPCs and DSCs were examined using the Cell-Light EdU Apollo 488 or 567 In Vitro Imaging Kit (RiboBio) as outlined by the manufacturer's protocol. Specifically, after incubation with 10 mM EdU for 2 hours, cells were fixed using 4% paraformaldehyde, permeabilized with 0.3% Triton X-100, and stained with Apollo fluorescent dyes. 5 μg/ml of DAPI was utilized to stain the cell nuclei for 10 minutes. The number of EdU-positive cells was assessed under a fluorescent microscope in five random fields. All assays were repeated independently three times.

Western blot analysis

Protein expression was evaluated by immunoblot analysis of cell lysates (20-40 μg) in RIPA buffer using rabbit antibodies to IGF-1 (1:1000; ZEN BIO, Chengdu, China), GAPDH, PI3K, p-PI3K, AKT, p-AKT(Ser473), MEK1/2, p-MEK1/2, ERK1/2 and AR (1:1000; CST, Danvers MA). The relative abundance of phosphorated proteins was determined by normalizing the levels with their corresponding total protein. The relative abundance of total protein was determined by normalizing the levels with their

corresponding endogenous control protein.

Supplementary Figures and Legends

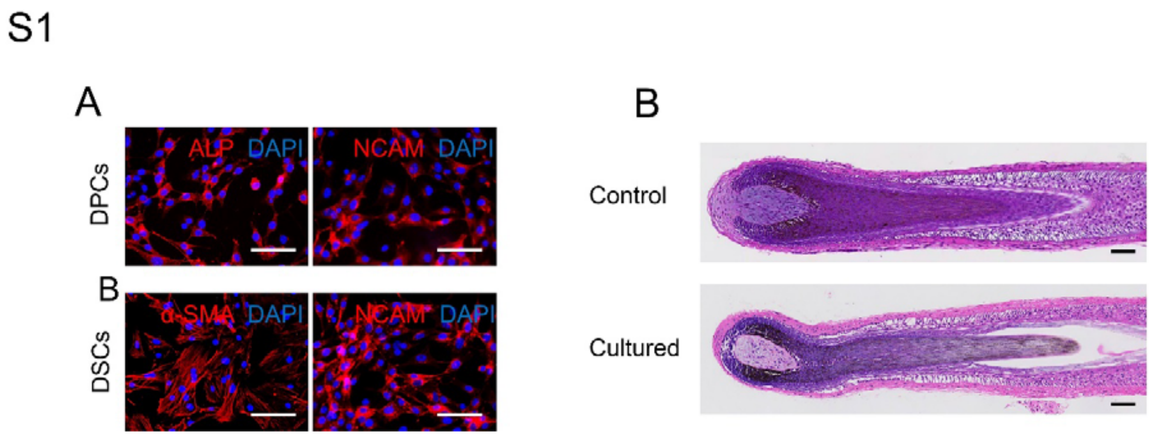

Figure S1. Identification of DPCs and DSCs, as well as the structure of cultured hair follicle. (A) Immunofluorescence experiments showed cultured DPCs and DSCs expressed its markers. (B) HE-staining showed cultured hair follicle maintained normal structure compared with fresh hair follicle. Scale bars represent 100 μm in (A&B).

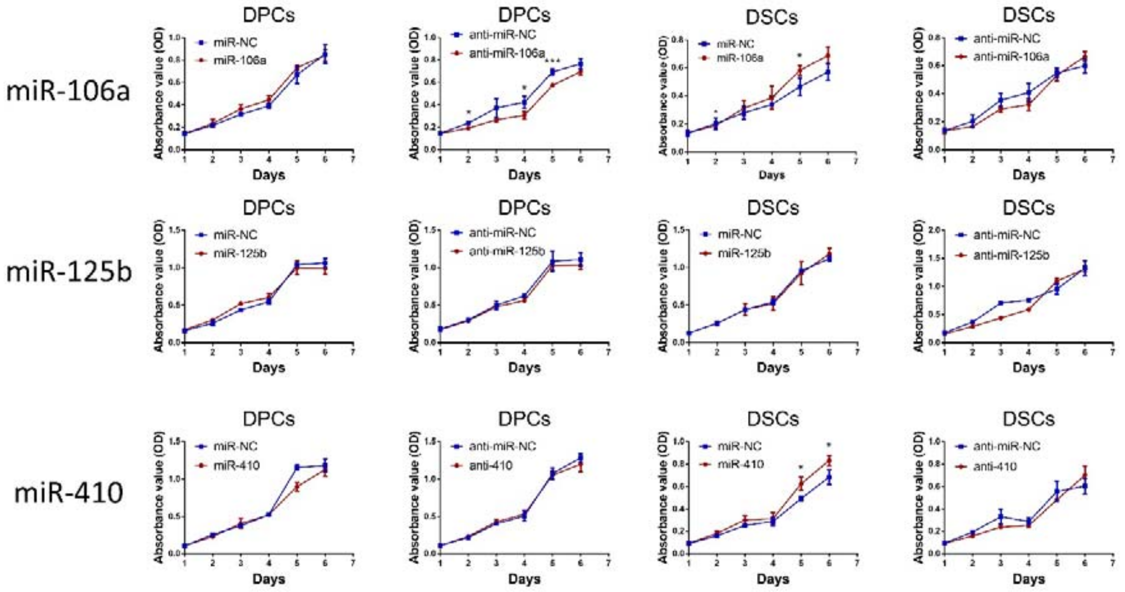

Figure S2. CCK-8 assays were performed to detect the effects of miR-106a, miR-125b and miR-410 on the proliferation of DPCs and DSCs, Student's t-test, mean ± SD, ****P*<0.05; ***P*<0.01; ****P*<0.001.

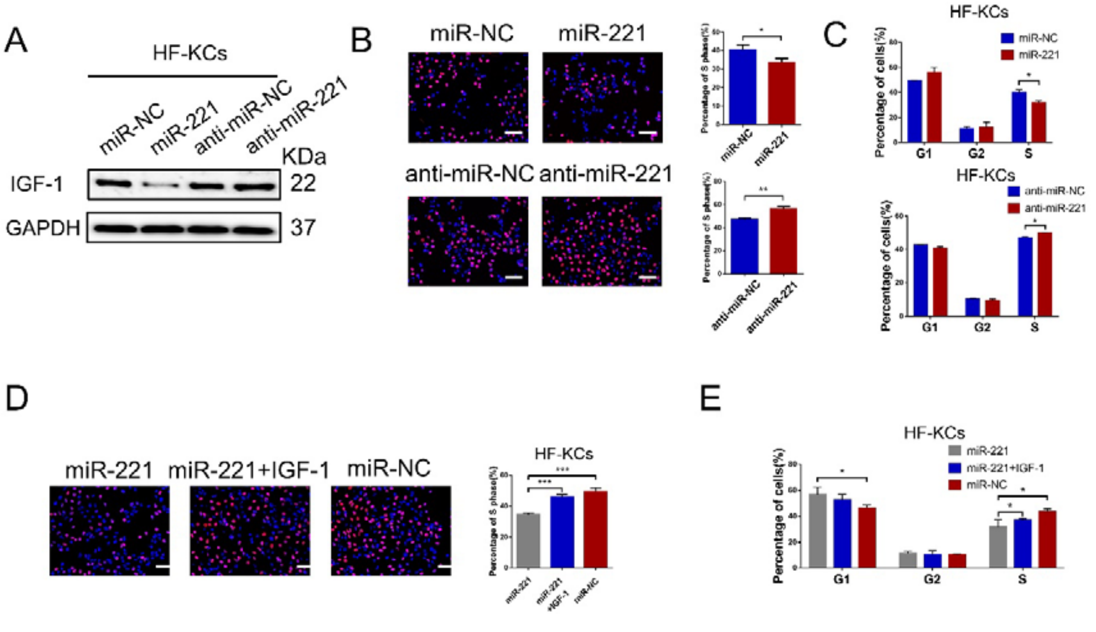

Figure S3. IGF-1 mediates the inhibitory effects of miR-221 on the proliferation of

HF-KCs. (A) Western blot analysis was performed to detect the protein expression of IGF-1 in HF-KCs transfected with miR-221 angomir or antagomir. EdU assay (B) and cell cycle assay (C) of HF-KCs were performed after transfected with miR-221 angomir or antagomir. EdU assay (D) and cell cycle assay (E) of HF-KCs were performed after treatments with anti-miR-NC, DHT + anti-miR-NC and DHT + anti-miR-221, Student's t-test, mean ± SD, **P* < 0.05; ***P* < 0.01; ****P* < 0.001. Scale bars represent 100 μm in (B&D).

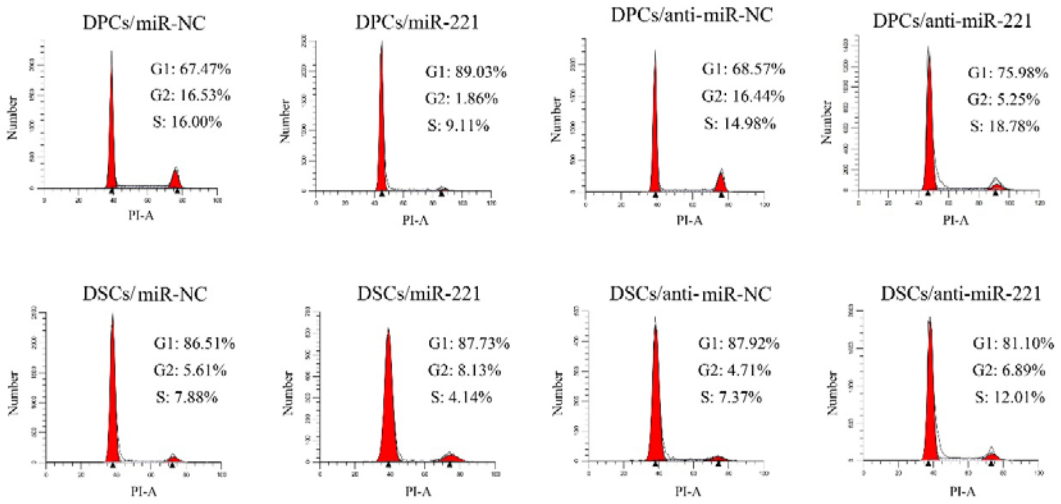

Figure S4. Cell cycle assays of DPCs and DSCs treated with miR-221 angomir and antagomir.

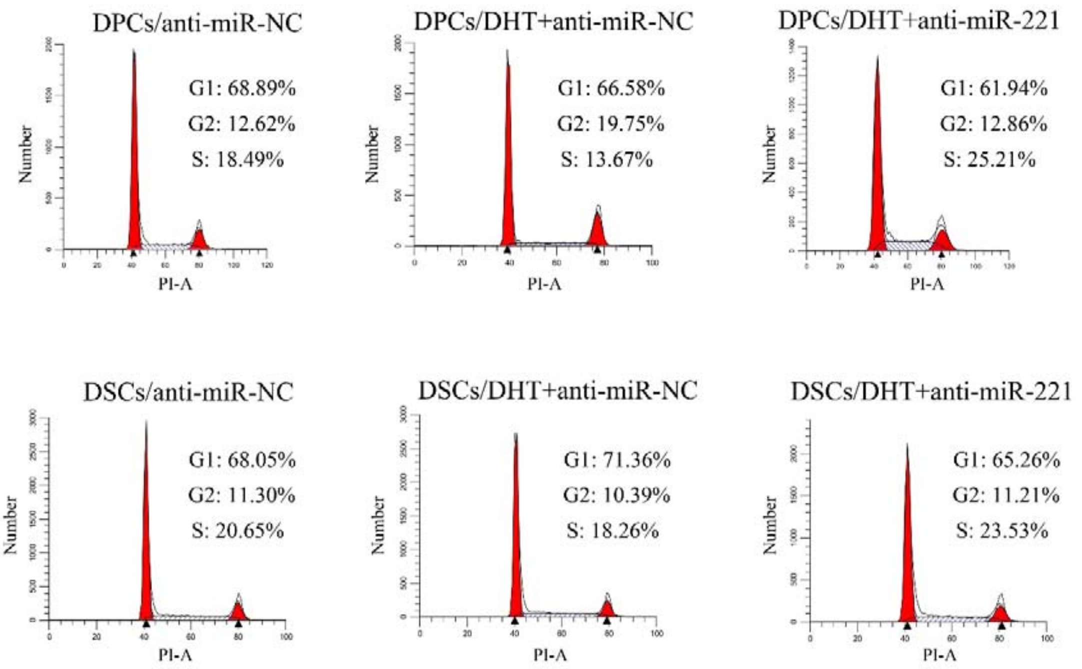

Figure S5. Cell cycle assays of DPCs and DSCs treated with anti-miR-NC, DHT + anti-miR-NC and DHT + anti-miR-221.

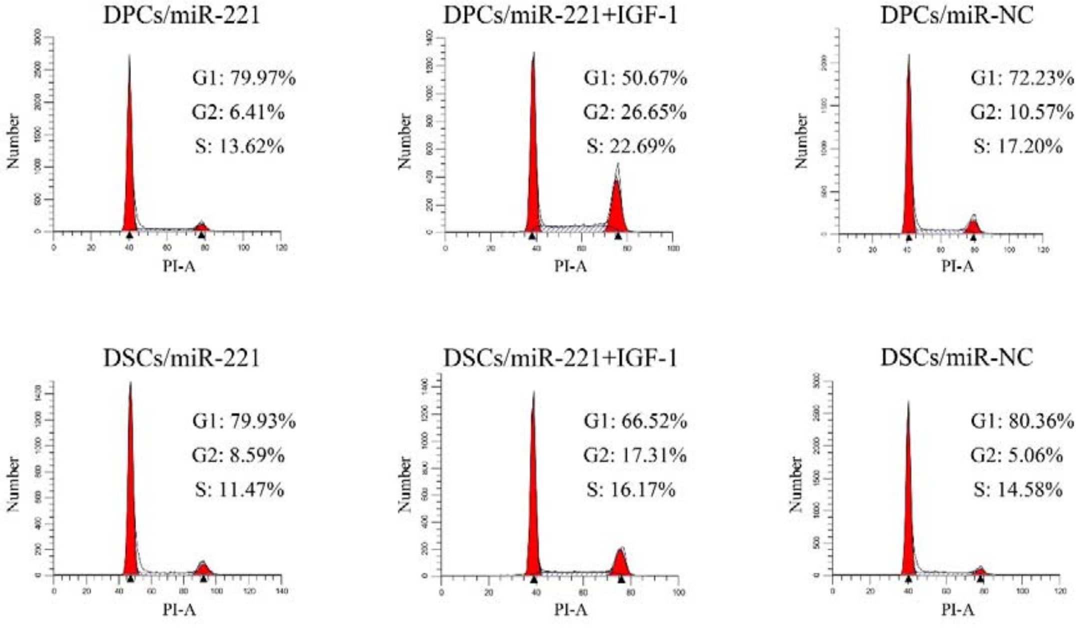

Figure S6. Cell cycle assays of DPCs and DSCs treated with miR-221, miR-221 + IGF-1 and miR-NC.

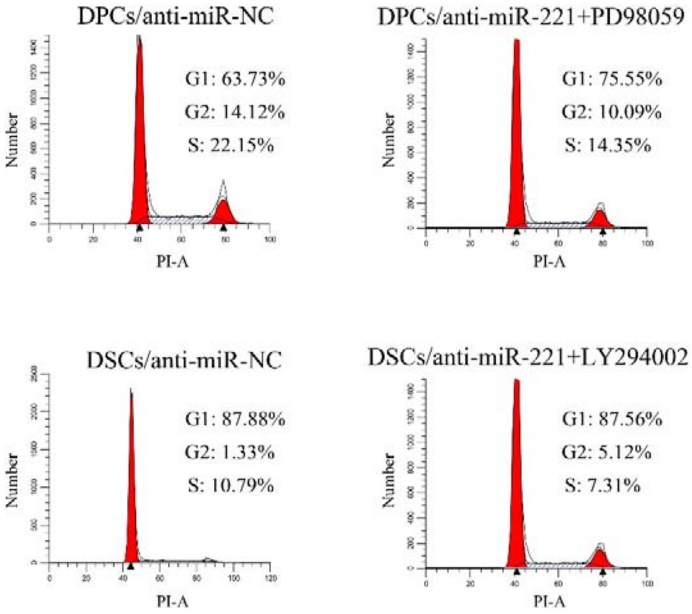

Figure S7. Cell cycle assays of DPCs treated with anti-miR-NC, anti-miR-221 + PD98059 and anti-miR-221, as well as DSCs treated with anti-miR-NC, anti-miR-221 + LY294002 and anti-miR-221.

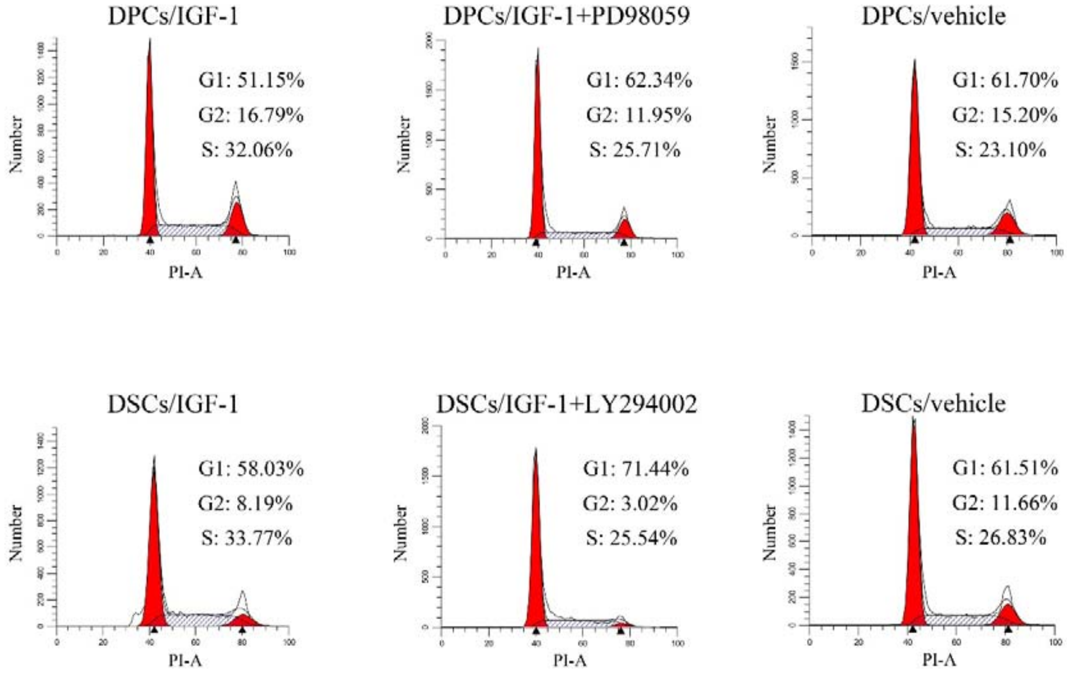

Figure S8. Cell cycle assays of DPCs treated with IGF-1, IGF-1 + PD98059 and vehicle, as well as DSCs treated with IGF-1, IGF-1 + LY294002 and vehicle.

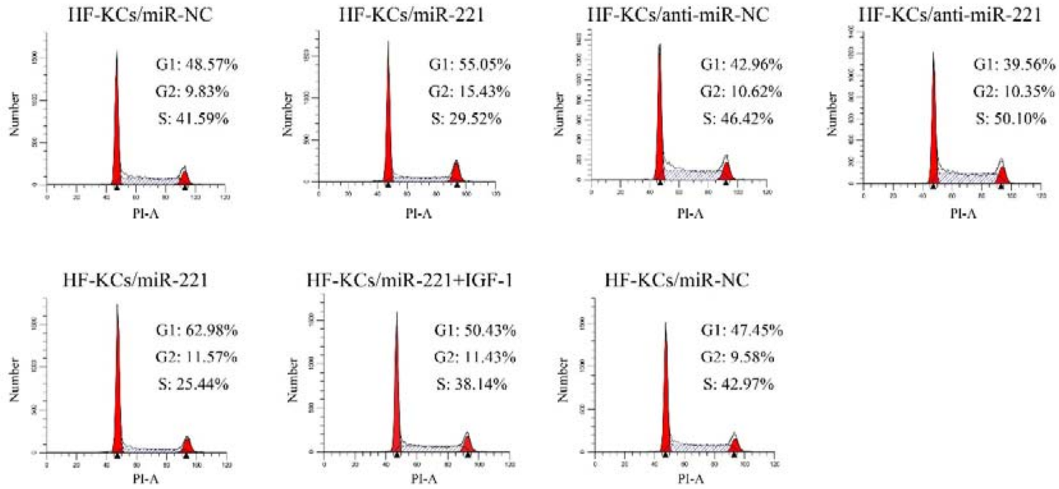

Figure S9. Cell cycle assays of HF-KCs treated with miR-221 angomir and antagomir, as well as HF-KCs treated with miR-221, miR-221 + IGF-1 and miR-NC.

Supplementary Table 1. q-PCR primer sequences for human genes

Gene name	Forward primer	Reverse primer
**IGF-1**	**GCTCTTCAGTTCGTGTGTGGA**	**GCCTCCTTAGATCACAGCTCC**
**AR**	**CCAGGGACCATGTTTTGCC**	**CGAAGACGACAAGATGGACAA**
**NRG1**	**ATGTGTCTTCAGAGTCTCCCAT**	**TGGACGTACTGTAGAAGCTGG**
**TRPC3**	**AGCAGCTCTTGACGATCTGG**	**GCACAACGAGACACTTGATAGC**
**KDR**	**GTGATCGGAAATGACACTGGAG**	**CATGTTGGTCACTAACAGAAGCA**
**CCN1**	**ACCGCTCTGAAGGGGATCT**	**ACTGATGTTTACAGTTGGGCTG**
**GRB10**	**CTCGTGGCAATGGATTTTTCTG**	**TCACTGTACTTAGGGTAGAAGGG**
**EGFR**	**AGGCACGAGTAACAAGCTCAC**	**ATGAGGACATAACCAGCCACC**
**CXCR5**	**CACGTTGCACCTTCTCCCAA**	**GGAATCCCGCCACATGGTAG**
**PDGFA**	**GCAAGACCAGGACGGTCATTT**	**GGCACTTGACACTGCTCGT**
**TIMP2**	**GCTGCGAGTGCAAGATCAC**	**TGGTGCCCGTTGATGTTCTTC**
**PAK1**	**CAGCCCCTCCGATGAGAAATA**	**CAAAACCGACATGAATTGTGTGT**
**GAB1**	**GATGGTTCGTGTTACGCAGTG**	**CGCTGTCTGCTACCAAGTAGAA**
**GAPDH**	**AAGGTCGGAGTCAACGGATTTG**	**CCATGGGTGGAATCATATTGGAA**

Supplementary Table 2. The sequence of IGF-1-3'UTR-wt and IGF-1-3'UTR-mut.

Name	Sequence
IGF-1-3'UTR-wt	CATTCACCCTAAGGATCCAATGGAATACTGAAAAGAAATCACTTCCTTGAAAATT<br>TTATTAAAAAACAAACAAACAAACAAAAAGCCTGTCCACCCTTGAGAATCCTTC<br>CTCTCCTTGGAACGTCAATGTTTGTGTAGATGAAACCATCTCATGCTCTGTGGCTC<br>CAGGGTTTCTGTTACTATTTATGCACTTGGGAGAAGGCTTAGAATAAAAGATGTA<br>GCACATTTTGCTTTCCCATTTATTGTTTGGCCAGCTATGCCAATGTGGTGCTATTGT<br>TTCTTTAAGAAAGTACTTGACTAAAAAAAAAAGAAAAAAAGAAAAAAAAGAAA<br>GCATAGACATATTTTTTTAAAGTATAAAAACAACAATTCTATAGATAGATGGCTTAA<br>TAAAATAGCATTAGGTCTATCTAGCCACCACCACCTTTCAACTTTTTATCACTCAC<br>AAGTAGTGTACTGTTCACCAAATTGTGAATTTGGGGGTGCAGGGGCAGGAGTTG<br>GA
IGF-1-3'UTR-mut	CATTCACCCTAAGGATCCAATGGAATACTGAAAAGAAATCACTTCCTTGAAAA<br>TTTTATTAAAAAACAAACAAACAAACAAAAAGCCTGTCCACCCTTGAGAATCC<br>TTCCTCTCCTTGGAACGTCAATGTTTGTGTAGATGAAACCATCTCATGCTCTG<br>TGGCTCCAGGGTTTCTGTTACTATTTATGCACTTGGGAGAAGGCTTAGAATA<br>AAAGcTcTcGaACATTTTGCTTTCCCATTTATTGTTTGGCCAGCTATGCCAAT<br>GTGGTGCTATTGTTTCTTTAAGAAAGTACTTGACTAAAAAAAAAAGAAAAAAA<br>GAAAAAAAAGAAAGCATAGACATATTTTTTTAAAGTATAAAAACAACAATTCT<br>ATAGATAGATGGCTTAATAAAATAGCATTAGGTCTATCTAGCCACCACCACCT<br>TTCAACTTTTTATCACTCACAAGTAGTGTACTGTTCACCAAATTGTGAATTTG<br>GGGGTGCAGGGGCAGGAGTTGGA

References:

1. Chen Y, Huang J, Liu Z, Chen R, Fu D, Yang L, et al. miR-140-5p in Small Extracellular Vesicles From Human Papilla Cells Stimulates Hair Growth by Promoting Proliferation of Outer Root Sheath and Hair Matrix Cells. Front Cell Dev Biol. 2020; 8: 593638.
2. Ohyama M, Zheng Y, Paus R, Stenn KS. The mesenchymal component of hair follicle neogenesis: background, methods and molecular characterization. Exp Dermatol. 2010; 19: 89-99.
3. Wen L, Miao Y, Fan Z, Zhang J, Guo Y, Dai D, et al. Establishment of an Efficient Primary Culture System for Human Hair Follicle Stem Cells Using the Rho-Associated Protein Kinase Inhibitor Y-27632. Front Cell Dev Biol. 2021; 9: 632882.
4. Chen Y, Huang J, Chen R, Yang L, Wang J, Liu B, et al. Sustained release of dermal papilla-derived extracellular vesicles from injectable microgel promotes hair growth. Theranostics. 2020; 10: 1454-78.
5. Kloepper JE, Sugawara K, Al-Nuaimi Y, Gáspár E, van Beek N, Paus R. Methods in hair research: how to objectively distinguish between anagen and catagen in human hair follicle organ culture. Exp Dermatol. 2010; 19: 305-12.
6. Kiuchi J, Komatsu S, Imamura T, Nishibeppu K, Shoda K, Arita T, et al. Low levels of tumour suppressor miR-655 in plasma contribute to lymphatic progression and poor outcomes in oesophageal squamous cell carcinoma. Mol Cancer. 2019; 18: 2.
7. Wang S, Kobeissi A, Dong Y, Kaplan N, Yang W, He C, et al. MicroRNAs-103/107 Regulate Autophagy in the Epidermis. J Invest Dermatol. 2018; 138: 1481-90.
8. Hu S, Li Z, Lutz H, Huang K, Su T, Cores J, et al. Dermal exosomes containing miR-218-5p promote hair regeneration by regulating β-catenin signaling. Sci Adv. 2020; 6: a1685.
9. Babapoor S, Horwich M, Wu R, Levinson S, Gandhi M, Makkar H, et al. microRNA in situ hybridization for miR-211 detection as an ancillary test in melanoma diagnosis. Modern Pathol. 2016; 29: 461-75.
